# Rethinking photosensitization in therapy and sun protection

**DOI:** 10.1039/d6sc01417h

**Published:** 2026-05-14

**Authors:** Erick L. Bastos, Waleska K. Martins, Rosangela Itri, Thiago T. Tasso, Maurício S. Baptista

**Affiliations:** a Department of Fundamental Chemistry, Institute of Chemistry, University of São Paulo 05508-000 São Paulo SP Brazil elbastos@usp.br; b Department of Biochemistry, Institute of Chemistry, University of São Paulo 05508-000 São Paulo SP Brazil baptista@iq.usp.br; c Instituto de Física, Universidade de São Paulo 05508-090 São Paulo SP Brazil; d Chemistry Department, Institute of Exact Sciences, Universidade Federal de Minas Gerais 31270-901 Belo Horizonte MG Brazil

## Abstract

Light-induced tissue damage involves photosensitization. This process forms the basis of photodynamic therapy but also underlies photoaging and skin cancer development during excessive sun exposure. This review bridges these interlinked, yet rarely connected fields. We propose a unified classification distinguishing photosensitizers, photocatalysts, photoinitiators, and photocatalytic initiators based on absorber fate and downstream radical chain propagation. This framework resolves longstanding ambiguities and carries direct mechanistic consequences. Our central thesis is that subcellular localization determines the biological outcome far more than quantum yields. Membrane-bound photosensitizers permeabilize organelles orders of magnitude more efficiently than their broadly distributed counterparts, provided direct photosensitizer-lipid contact favors truncated lipid generation through Type I electron-transfer reactions rather than singlet oxygen oxidation. Organelle-specific targeting exploits this principle. Mitochondrial photodamage triggers regulated cell death through cardiolipin oxidation and cytochrome c release. Lysosomal targeting induces cathepsin release and autophagy dysfunction, often proving more effective for long-term cell killing. Dual-organelle strategies activate apoptosis, ferroptosis, and pyroptosis synergistically. These same mechanisms operate during sun exposure. Lipofuscin mediates radical chain amplification, creating a feed-forward cycle of visible light sensitivity in aging skin. Pheomelanin acts as a Type I photosensitizer generating superoxide and hydrogen peroxide under visible light, while eumelanin dissipates photon energy as heat. The amplification inherent in photoinitiated processes explains why modest light doses overwhelm antioxidant defenses, tipping redox homeostasis from eustress to distress. We translate these insights into mechanistically rational photoprotection strategies addressing visible light beyond conventional UV filters and into design principles for next-generation therapeutic photosensitizers.

## Abstract 2

Five functional modes of photosensitized reactions show that absorber localization, not quantum yield, decides whether chain propagation amplifies oxidative damage and depletes reducing equivalents, linking photodynamic therapy to solar skin damage.

## Graphical abstract

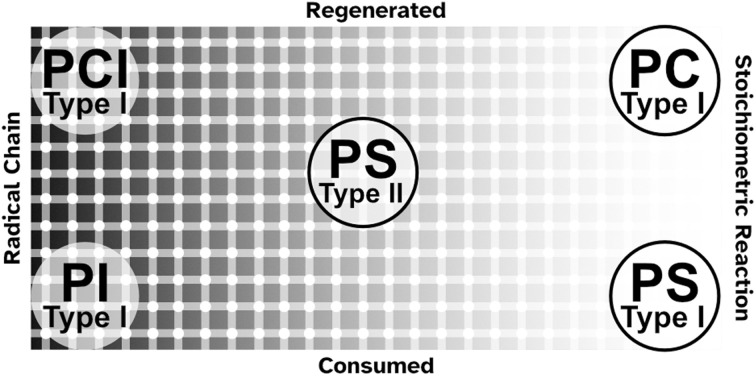

## 1 Introduction

Photodynamic therapy (PDT) deliberately kills cells by generating oxidants at specific subcellular sites.^1,2^ Sunscreen technology exists to prevent exactly this process. Despite sharing identical photochemistry (a chromophore absorbs light, populates excited states, and generates reactive species that oxidize biomolecules), these two fields have developed in almost complete isolation. PDT research seeks to optimize photosensitizer localization and reactivity to maximize cell death, while suncare filters minimize photon delivery to skin. Yet neither field has systematically drawn on the other's mechanistic insights. This review argues that bridging this gap reveals principles with transformative implications for both disciplines.

PDT is a sequential process involving three core components: a photosensitizer, light of a specific wavelength (typically 600 to 1000 nm), and oxygen. The therapy begins with the selective incorporation and localization of the photosensitizer in diseased tissue. When exposed to light, the photosensitizer is activated and triggers photochemical reactions that consume oxygen, leading to photosensitized oxidation reactions.^2–4^ These reactions produce biological responses such as direct cell death and stimulation of the immune system, ultimately destroying the targeted tissue. The potential of PDT for treating a variety of diseases is significant and multifaceted. The literature provides numerous examples of successful clinical applications, from melanoma and Kaposi's sarcoma to diabetic foot osteomyelitis, onychomycosis, and neglected tropical diseases like leishmaniasis.^2,5–11^ This versatility suggests that a single, relatively simple technology platform could address numerous pressing health concerns. Despite its unique advantages, such as exceptional tumor selectivity, minimal invasiveness, and repeatable treatment without cumulative resistance, PDT remains a peripheral modality in clinical oncology, rarely a first-line option and often overlooked in medical curricula. The critical bottleneck lies in the photosensitizers themselves, which largely rely on outdated porphyrin and chlorin chemistry from the 1990s.^12^

The knowledge gap is symmetrical. Current sunscreens are designed to absorb or scatter UV radiation, even though visible light also contributes to skin damage. High energy visible light from 400 to 500 nm penetrates deeper into the dermis, while UV radiation is mainly absorbed in the epidermis.^13,14^ Keratinocytes, melanocytes, and fibroblasts all contain endogenous photosensitizers such as protoporphyrin IX, lipofuscin, flavins, and pheomelanin, which are concentrated in mitochondria, lysosomes, and melanosomes.^15^ When these molecules are photoexcited, they generate oxidants that damage mitochondrial DNA, drive lipid peroxidation, and impair electron transport chain function. This mechanism contributes to photoaging, with both dermal and epidermal cells developing mitochondrial and lysosomal dysfunction despite exposure to lower energy photons than UV irradiated epidermal cells.^16–18^ Research in photodynamic therapy has shown that photosensitizer localization, membrane contact, and organelle targeting determine biological outcomes. These principles are well established in that field, but they have not been systematically applied to photoprotection strategies.

Our central thesis is that the subcellular localization of a photosensitizer determines the biological outcome far more than its photochemical efficiency. This principle, established through decades of PDT research comparing photosensitizers of vastly different quantum yields, has direct and unexploited implications for how we understand and prevent light-induced skin damage. If localization matters more than yield, then photoprotection strategies should focus not only on blocking photons but also on preventing photosensitizer accumulation at sensitive subcellular sites and interrupting the contact-dependent reactions that drive membrane permeabilization.

This review bridges the traditionally separate fields of PDT and suncare by examining their shared mechanistic foundations. We begin by clarifying the relationship between photosensitization and photocatalysis (Section 2), concepts frequently conflated in the literature, and propose a unified classification that eliminates ambiguity across disciplines. We then examine the emerging paradigm that photosensitizer localization overrides photochemical efficiency as the primary determinant of a biological outcome (Section 3). The molecular mechanisms of membrane photodamage are discussed in detail, with emphasis on why contact-dependent Type I reactions, rather than diffusible singlet oxygen (^1^O_2_, ^1^Δ_g_), drive membrane permeabilization (Section 4). Organelle-specific photodamage responses, including mitochondrial, lysosomal, and dual-targeting strategies, are analyzed in the context of regulated cell death pathways (Section 5). We then address the cellular redox homeostasis framework within which photodamage operates, distinguishing oxidative eustress from distress (Section 6). The endogenous photosensitizers responsible for cutaneous photodamage, including lipofuscin, melanin, and flavins, are examined through the same mechanistic lens developed for PDT (Section 7). Finally, we translate these insights into a framework for next-generation photoprotection strategies that address visible light beyond conventional UV filters (Section 8).

## 2 Reframing photosensitization

A light-absorbing species that drives a chemical reaction in another molecule may be referred to as a photosensitizer, photocatalyst, or photoinitiator, depending on subtle mechanistic differences and disciplinary context.^19–22^ This section systematizes the classification of indirect photoinduced processes in biological environments by clarifying the defining criteria that distinguish each role. The framework developed here is not merely taxonomic. As subsequent sections will show, these distinctions carry direct mechanistic consequences for understanding membrane photodamage, organelle-specific cell death, and cutaneous photobiology.

### 2.1 The key problem: overlapping definitions

Photosensitizers, photocatalysts, and photoinitiators all enable photochemical reactivity. While extreme cases are straightforward to classify, labeling borderline examples can be challenging. The central ambiguity arises from the requirement, or lack thereof, for regeneration of the light-absorbing species and the diversity of photosensitizer fates.^23^ This is further complicated by the fact that many species referred to as catalysts are actually activators,^24,25^ species that promote reactions but are consumed in the process, as in the case of base-promoted ester hydrolysis.^26^ To reconcile these inconsistencies and eliminate ambiguity while enabling clear classification of phenomena across different environments, we propose a reframing of these roles.

A photosensitizer (PS) is a species that absorbs light on behalf of another molecular entity and, through an excited-state process, induces a physical or chemical change in that entity. The photosensitizer acts as a mediator because it converts photon energy into a form of activation that the target species cannot achieve by direct irradiation under the same conditions. The interaction may proceed *via* energy transfer, electron transfer, hydrogen atom transfer, or other mechanisms. Light absorption by a photosensitizer is necessary for the occurrence of the transformation, but PS regeneration is not required. This constitutes a deliberate departure from the IUPAC definition of photosensitization, which stipulates that, in mechanistic photochemistry, the photosensitizer is not consumed in the reaction.^27^ However, this strict requirement renders the photosensitizer and photocatalyst nearly synonymous, since both demand regeneration and both mediate the transformation of another species upon light absorption. Indeed, the IUPAC Glossary of Photocatalysis states that “*should the sensitiser be recycled in the overall process, then the photosensitiser is acting as a photocatalyst*”.^24^ This leaves the field with two terms for recycled species and no umbrella term for light-absorbing mediators regardless of their fate and the nature of the downstream process. In a previous analysis of photosensitization reactions of biomolecules, Baptista and coauthors noted that many compounds widely accepted as photosensitizers are consumed in the photochemical process, and argued for a more extensive and pragmatic definition.^28^ The present framework formalizes this observation: removing the regeneration requirement allows the photosensitizer to serve as the general category under which more specific functional roles are defined.

A photocatalyst (PC) is a photosensitizer that takes part in a catalytic cycle to accelerate a chemical transformation and is regenerated per turnover. Two criteria are essential: (i) the photosensitizer must itself undergo a chemical change during the productive cycle, distinguishing photocatalysis from processes in which the sensitizer mediates only a physical step such as energy transfer, and (ii) it must be regenerated as an integral part of the productive mechanism. Photobleaching and degradation through competing, non-productive pathways do not alter the classification. This converges with the notion that all photocatalysts are photosensitizers, but not all photosensitizers are photocatalysts. Notably, the two roles differ in kind, not just degree. In thermal catalysis, the catalyst accelerates a thermodynamically accessible transformation by lowering the activation barrier. A photosensitizer, by contrast, enables access to an otherwise inaccessible reaction coordinate as the excited state opens a pathway that simply does not exist in the dark. When regeneration and turnover are added, the photosensitizer also acts as a catalyst, but the enabling function remains primary. This distinction underpins the proposed classification.

A photoinitiator (PI) falls within the broad definition of a photosensitizer but is consumed in the process of generating radicals or other reactive intermediates that initiate a chemical chain reaction. The depletion of the photoinitiator is the productive step itself. This distinguishes photoinitiators from photosensitizers that are merely degraded through incidental photobleaching. The distinction is mechanistic, not merely kinetic, because the photoinitiator's consumption is integral to the initiation event, not a side reaction. A different situation arises when the photosensitizer operates in a closed redox cycle and regenerates per turnover while producing radicals or other reactive intermediates that initiate chain reactions. IUPAC encompasses both rate enhancement and initiation under the single term photocatalysis.^27^ However, we term this species a photocatalytic initiator (PCI) because the stoichiometric relationship between absorber turnovers and product molecules is fundamentally different from that of a photocatalyst. In a PC, each product molecule ideally requires one catalytic turnover, whereas in a PCI, one turnover generates a radical that can yield many product molecules *via* chain propagation. This concept is well established in polymer chemistry, where multicomponent formulations based on a dye, an electron acceptor, and an electron donor are designated photocyclic initiating systems (PCISs).^29,30^ While PCIS refers to the multicomponent system, PCI as defined here refers to the functional role of the light-absorbing species within it. This concept was demonstrated explicitly for Rose Bengal. In the presence of a triazine acceptor, Rose Bengal is consumed after approximately 5 photocycles, functioning as a photoinitiator. When a tertiary amine is added to the system, the amine reduces the oxidized dye back to its ground state, closing the catalytic cycle. Under these conditions, Rose Bengal survives more than 210 photocycles before bleaching, directly quantifying the catalytic turnover that distinguishes PCI from PI behavior.^30^ Kinetic modeling of the three-component system showed that dye regeneration, additional radical production, and consumption of terminating photoproducts are mechanistically intertwined, providing direct kinetic evidence for the dual catalytic-and-initiating nature of the PCI role.^31^ Indeed, eosin, Rose Bengal, fluorescein, and riboflavin all exhibit cyclic dye regeneration during photoinitiation, a behavior that enhances initiation kinetics by continuously producing radicals while the dye survives.^32^ We propose that this concept, developed and refined in the context of photopolymerization, is directly applicable to biological photosensitized transformations, where chain reactions are common and the fate of the primary absorber is often ambiguous.

A central feature of this framework is that it classifies mechanistic roles, not molecules. The same compound can occupy different categories depending on the reaction partner, the medium, and the experimental conditions, because turnover is not an intrinsic property of a light-absorbing species but an engineered outcome that depends on the availability of suitable cycle-closure agents. An absorber that operates as a photocatalyst in the presence of a co-reductant may function as a photoinitiator in its absence. Two boundary cases test this principle. The first concerns the distinction between productive consumption and photobleaching. In many biological systems, the reactive species generated by a photosensitizer, including singlet oxygen, superoxide, and peroxyl radicals, can react back with the primary absorber and destroy it. This does not alter its functional classification. The absorber is not depleted to create the reactive species but is destroyed because of them. Its loss is collateral damage through a competing, non-productive pathway, not the mechanistically integral step that generates the reactive intermediate. A porphyrin that produces singlet oxygen *via* energy transfer and is subsequently oxidized by that same singlet oxygen remains a photosensitizer. A pterin running a closed photoredox cycle with tryptophan that is eventually destroyed by accumulated superoxide remains a photocatalyst. The classification is determined by what is mechanistically integral to the productive pathway, not by the net fate of the absorber after prolonged irradiation. The second concerns autocatalytic photosensitization, where the photosensitized reaction generates a product that itself functions as a photosensitizer for the same transformation. The original species may be consumed, classifying it as a PI at the molecular level, while the system exhibits catalytic behavior at the network level. Because this framework classifies species at the molecular level, the original absorber is a photoinitiator if its consumption is integral to the productive pathway, regardless of the emergent behavior of the product network.

Finally, the present classification complements existing approaches that address different aspects of the photocatalytic mechanism. Capaldo and Ravelli classified photocatalytic processes according to the identity of the species responsible for regenerating the exhausted photocatalyst, whether a reaction intermediate, a co-catalyst, a reaction partner, an electrode, or other agents, demonstrating that the nature of the cycle-closure step can decisively influence reaction selectivity and outcome.^23^ Their analysis asks how the photocatalyst is regenerated. Ours asks what functional role the light-absorbing species plays. Together, they provide a more complete description of any photoinduced transformation. By resolving these definitional overlaps, this classification provides a consistent basis for interpreting photoinduced transformations in biological environments, where mechanistic ambiguity is the norm rather than the exception.

### 2.2 Classifying photoinduced biochemical transformations

**Fig. 1:**
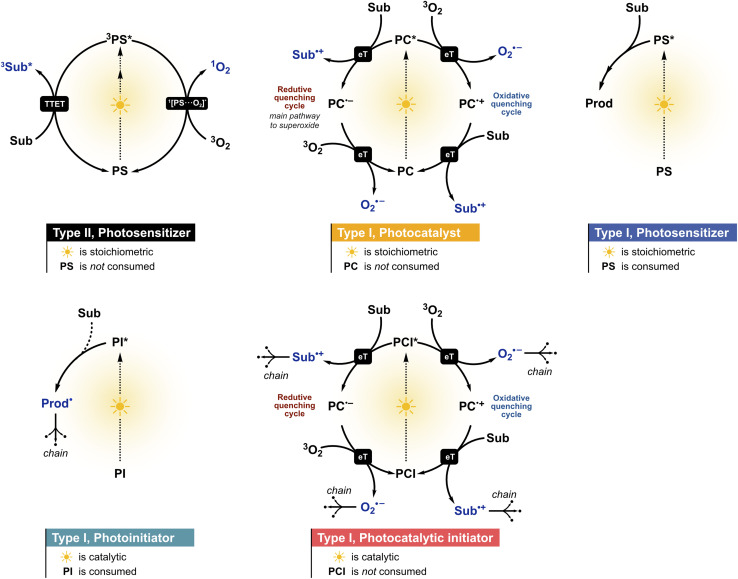
Mechanistic schemes for the functional classification of light-absorbing species in photoinduced oxidations of biological interest. The same compound can occupy different categories depending on the reaction partner, medium, and conditions (see the text). Photobleaching through non-productive pathways does not alter the classification. The label eT encompasses both single electron transfer (SeT) and proton-coupled electron transfer (PCeT), including hydrogen atom transfer (HAT); reactions were drawn as SeT for clarity. Species in navy blue are the oxidizing agents of interest. TTET = triplet–triplet energy transfer and Sub = substrate. Dashed lines indicate that species may or may not participate.

Type I and Type II photosensitized oxidations classify how the overall reaction is triggered, by electron or hydrogen atom transfer or by energy transfer to molecular oxygen producing singlet oxygen, respectively.^33^ Neither designation captures the functional role of the light-absorbing species. The classification proposed here rests on whether the absorber is consumed or regenerated, and whether the downstream process is stoichiometric or chain-propagating. These mechanistic categories are distinct from functional role-based classifications such as PS, PC, PI, and PCI, but not independent of them, since the mechanism of activation constrains which roles are accessible to the primary absorber. Accordingly, a given transformation should be described by the mechanism label (Type I or Type II), that identifies the photosensitized oxidation process, and the role label (PS, PC, PI, or PCI) that refers to the absorber (Fig. 1).

**Fig. 2:**
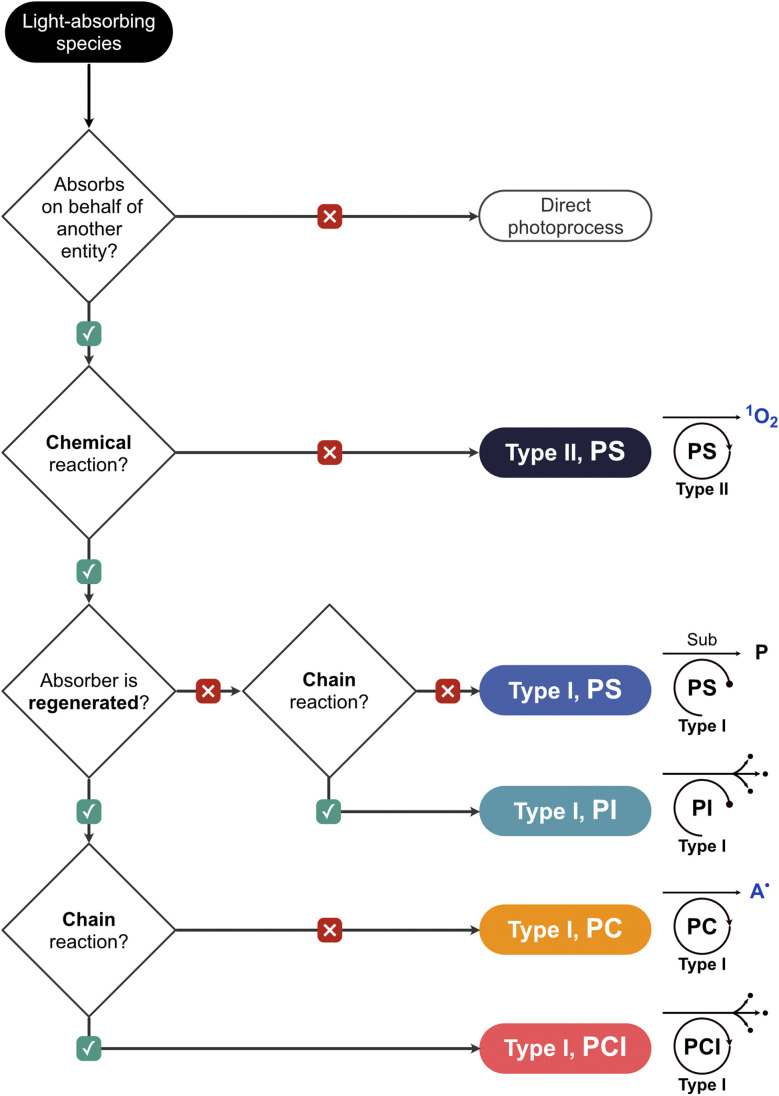
Classification of light-absorbing species in photoinduced biological transformations. Decision tree for assigning the functional role of a PS based on two criteria: whether the absorber is regenerated during the productive cycle and whether the downstream process is stoichiometric or chain-propagating. Colors and symbols are used consistently throughout the text. A˙ represents a generic radical, including superoxide.

A Type II process is inherently physical at the level of the primary absorber, since energy transfer returns the photosensitizer to its ground state without chemical transformation, and therefore the absorber always functions as a photosensitizer. This classification holds because the productive step, energy transfer to O_2_, does not chemically alter the absorber, and any subsequent destruction of the absorber by the singlet oxygen it generated constitutes non-productive photobleaching (as discussed in Section 2.1). A Type I transformation, on the other hand, involves a chemical change at the primary absorber through electron or hydrogen atom transfer. The absorber may still function as a photosensitizer if no net chemical transformation results, but this mechanism also opens the possibility of the absorber acting as a photocatalyst if a closed redox cycle regenerates it per turnover, as a photoinitiator if it is irreversibly consumed to generate chain-propagating radicals, or as a photocatalytic initiator if it is regenerated in a closed cycle while the radicals produced propagate a chain independently. The systematic basis proposed here for interpreting photoinduced transformations in biological systems is necessary, as the following sections will demonstrate, for discussing why the localization of an absorber within a cell matters far more than how efficiently it generates any single reactive species. A decision flowchart is depicted in Fig. 2 and the examples from biological systems in **Box 1** illustrate how these two axes of classification operate in practice.

**Box 1. Classification of selected photoinduced processes in biological systems according to the mechanism and functional role.**

**Porphyrins in photodynamic therapy**: Type II/photosensitizer. Porphyrin derivatives such as hematoporphyrin and its clinical analogues (porfimer sodium/photofrin, and temoporfin/foscan) are the cornerstone of photodynamic therapy. Upon irradiation, their triplet excited state transfers energy to ground-state molecular oxygen, generating ^1^O_2_.^34,35^ The singlet oxygen then reacts with cellular targets, mediating oxidative damage to cancer cells. Under the present framework, the porphyrin is classified as a photosensitizer.

**Phthalocyanine PDT** (*e.g.*, silicon phthalocyanine 4): Type II/photosensitizer. Phthalocyanines and their metallated derivatives, including silicon phthalocyanine 4 (Pc4) currently in clinical PDT trials for cutaneous T-cell lymphoma, generate singlet oxygen *via* energy transfer from the triplet excited state to ground-state O_2_ with quantum yields (*Φ*_Δ_ ≈ 0.4–0.7) typical of efficient Type II photosensitizers.^36^ The productive molecular step returns the absorber to the ground state without net chemical alteration, regardless of how strongly the cellular outcome depends on subcellular localization. Phthalocyanines are therefore classified as photosensitizers within the present framework; subsequent photobleaching by the ^1^O_2_ they generate is a non-productive side pathway and does not alter the role assignment.

**Pterin-sensitized oxidation of tryptophan**: Type I/photocatalyst. Pterins are endogenous heterocyclic compounds that accumulate in the depigmented skin of vitiligo patients and act as efficient photosensitizers under UV-A irradiation.^37^ Irradiation of pterin in aerated aqueous solutions containing tryptophan leads to oxidation of the amino acid, while the pterin concentration remains unchanged.^38^ The mechanism proceeds *via* electron transfer from tryptophan to triplet-excited pterin, generating the pterin radical anion and the tryptophan radical cation. Molecular oxygen then reoxidizes the pterin radical anion, regenerating the ground-state photosensitizer and producing superoxide (O_2_˙^−^).^39^ The primary absorber undergoes a chemical transformation (one-electron reduction) and is regenerated through a closed redox cycle: each tryptophan molecule oxidized requires one pterin redox turnover. Pterin in this context functions as a photocatalyst operating *via* a Type I mechanism.

**Pterin–thymine adduct formation**: Type I/photosensitizer (consumed without chain initiation). Under anaerobic conditions, the photochemistry of pterin with thymine nucleotides takes a fundamentally different course. Electron transfer from thymine to triplet-excited pterin generates a radical pair that, instead of undergoing back electron transfer, recombines to form a covalent pterin–thymine adduct.^40,41^ Pterin is irreversibly consumed and the reaction does not generate radicals that propagate a chain. Instead, each adduct formed requires one pterin molecule consumed. This places pterin as a PS, as pterin absorbs light on behalf of thymine and induces a chemical change.

**Riboflavin-mediated protein photooxidation**: Concurrent Type I/photocatalyst and Type II/photosensitizer. Riboflavin (vitamin B2) is an endogenous photosensitizer that mediates photooxidation of proteins through both Type I and Type II pathways.^42,43^ In the Type I pathway, the triplet state of riboflavin accepts an electron from susceptible amino acid residues (tryptophan, tyrosine, and histidine), forming the riboflavin radical anion, which is subsequently reoxidized by molecular oxygen in a closed redox cycle.^15^ Each amino acid residue oxidized requires one riboflavin redox turnover. In parallel, triplet riboflavin transfers energy to molecular oxygen, generating singlet oxygen that independently oxidizes protein targets. Riboflavin therefore operates simultaneously as a photocatalyst and a Type II photosensitizer, playing two functional roles at once.

**Lipid peroxidation**: Type I/photoinitiator or/photocatalyst initiator. When an excited absorber abstracts a hydrogen atom from an unsaturated lipid, it generates a carbon-centered lipid radical (L˙) that reacts with O_2_ to form a peroxyl radical (LOO˙).^44,45^ This peroxyl radical abstracts hydrogen from an adjacent lipid, yielding a hydroperoxide and regenerating the chain carrier, establishing self-propagating lipid peroxidation.^46^ If the absorber is consumed in the initiation step, it functions as a PI; if a redox cycle regenerates it, it functions as a PCI.

**Riboflavin-sensitized lipid peroxidation**: Type I/photocatalytic initiator. Under aerobic conditions, energy transfer from triplet-excited riboflavin to molecular oxygen (Type II) is kinetically favored over direct reaction with lipids, making singlet oxygen the dominant oxidant of unsaturated lipids in most biological settings.^47^ When riboflavin is embedded in or closely associated with a lipid membrane, however, the contact-dependent pathway becomes competitive and triplet-excited riboflavin abstracts a hydrogen atom from an unsaturated lipid, generating a lipid radical and a semireduced riboflavin radical.^44,47^ The semireduced riboflavin radical is then reoxidized by molecular oxygen, regenerating the ground-state absorber and producing superoxide as a byproduct, thereby closing the catalytic cycle. The lipid radical, meanwhile, enters the same self-propagating chain described above, reacting with O_2_ to form a peroxyl radical that abstracts hydrogen from the next lipid, yielding a hydroperoxide and a new lipid radical. One riboflavin turnover therefore generates many lipid hydroperoxide molecules *via* chain propagation, and riboflavin survives to initiate further chains. This is a PCI operating *via* a Type I mechanism. Similarly, any absorber that catalytically generates superoxide through a closed Type I redox cycle can act as a PCI if the superoxide dismutates to H_2_O_2_, which undergoes Fenton chemistry to produce hydroxyl radicals that propagate chain oxidation of biomolecules.^33,48^

**Photooxidation of NADH in cells**: Type I/photocatalyst. Recent work has demonstrated that well-known photosensitizers used in photodynamic therapy, including Rose Bengal, BODIPY derivatives, phthalocyanines, and porphyrins, can catalyze the oxidation of the cellular electron carrier NADH to NAD^+^ under irradiation.^49^ In this process, the excited photosensitizer oxidizes NADH *via* single-electron transfer and is subsequently regenerated by transferring the electron to a cellular acceptor, completing a closed photoredox cycle. Each NADH molecule converted requires one redox turnover of the photosensitizer, and no chain reaction is involved. These findings suggest that the role of the same porphyrin derivative in a cellular context may be simultaneously that of a photosensitizer (*via* Type II energy transfer to O_2_) and a photocatalyst (*via* Type I photoredox cycling of NADH), further reinforcing the independence of the mechanistic and role-based classification axes.

A note on terminology: the collective terms “reactive oxygen species” (ROS) and “reactive nitrogen species” (RNS) remain widespread but obscure critical mechanistic distinctions. Singlet oxygen, superoxide, hydroxyl radicals, and hydrogen peroxide differ by orders of magnitude in reactivity, selectivity, and diffusion distance. Following recent consensus recommendations,^50,51^ this review names specific oxidants wherever the chemistry is established and uses “oxidants” as the general term when multiple species are involved. This choice is not merely stylistic, as the framework developed above classifies photosensitized processes according to the type of reactive intermediate produced, making specificity essential.

## 3 The localization paradigm: why position matters more than yield

The prevailing paradigm in PDT links photosensitizer efficacy to singlet oxygen quantum yield (*Φ*_Δ_), directing synthetic efforts toward maximizing photochemical output.^52–54^ This perspective is incomplete.^7,55^ Subcellular localization, not photochemical efficiency, is the dominant determinant of phototoxic outcome, and the underlying reasons are more nuanced than a simple comparison of *Φ*_Δ_s would suggest.

### 3.1 The methylene blue paradox

**Fig. 3:**
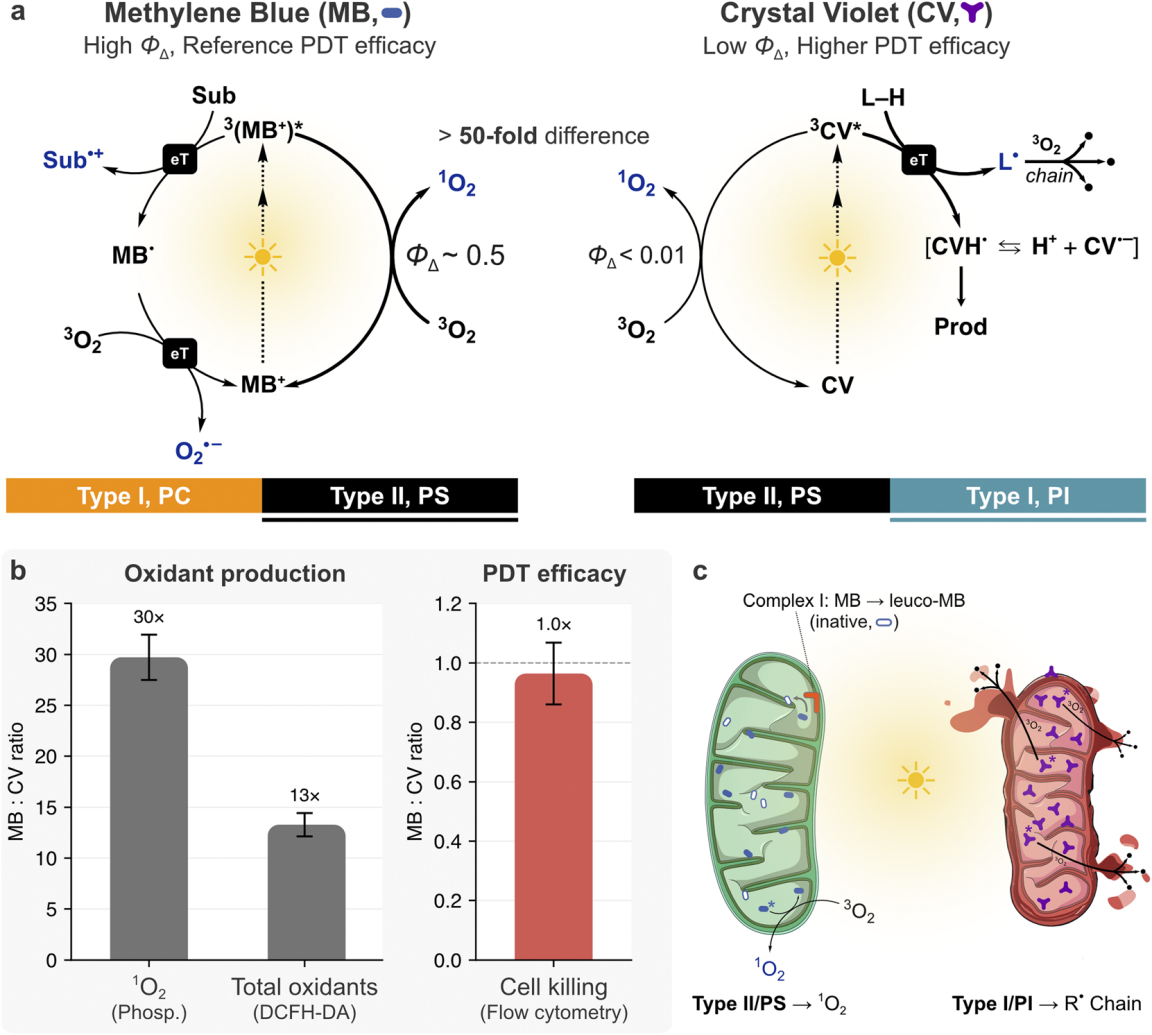
The methylene blue paradox. (a) Photochemical mechanisms of MB and CV. MB generates ^1^O_2_ through energy transfer and also undergoes electron-transfer pathways producing substrate radicals and superoxide, while being regenerated (Type I, PC). CV produces negligible ^1^O_2_ but, when conformationally restricted at membranes, abstracts hydrogen atoms from lipids, generating lipid radicals that propagate peroxidation chains (Type I, PI). The >50-fold difference in singlet oxygen quantum yield predicts a proportional disparity in phototoxicity that is not observed. (b) MB:CV ratio for oxidant production and PDT efficacy in HeLa cells at 20 µM (data replotted from Oliveira *et al.*^58^). MB produces 30-fold more ^1^O_2_ and 13-fold more total oxidants, yet cell killing is equivalent. (c) Biological basis of the paradox at mitochondria. Complex I reduces MB to the colorless, photodynamically inactive leuco-MB, depleting the active absorber at its primary accumulation site. CV (purple) resists stable two-electron reduction, remains embedded in the inner mitochondrial membrane, and generates radicals that propagate lipid peroxidation along cardiolipin-rich cristae.

We set out to test whether subcellular localization matters more than photochemical yield by comparing methylene blue (MB, *Φ*_Δ_ ≈ 0.5; *ε*_664 nm_ ≈ 85 000 M^−1^ cm^−1^),^56^ an efficient ^1^O_2_ generator, with Crystal Violet (CV, *Φ*_Δ_ < 0.01; *ε*_590 nm_ ≈ 75 800 M^−1^ cm^−1^),^57,58^ expecting that MB would induce far greater photoinduced cell death (Fig. 3a). Despite its superior photochemistry in water and less polar media,^59^ MB was consistently outperformed by CV in photodynamic cell killing, even after subtracting dark toxicity and normalizing for absorbed photons (Fig. 3b).^58^

#### 3.1.1 MB is inactivated at its own target

As a lipophilic cation, MB accumulates up to ∼1000-fold in the mitochondrial matrix,^60^ which is a highly reducing environment^61^ compared to the intermembrane space and cytosol. The low redox potential of MB [E°′(MB^+^/leuco-MB) = +11 mV] makes it an excellent substrate for Complex I, which reduces it to leucomethylene blue (leuco-MB), a colorless species with no photodynamic activity (Fig. 3c).^60,62^ Energized mitochondria (*i.e.*, with active electron transport chains) generate approximately half the ^1^O_2_ of uncoupled controls, because NADH-driven reduction of MB to leuco-MB depletes the photosensitizer pool.^60^ In HeLa cells, MB was essentially fully reduced and invisible, while the structurally similar toluidine blue, which resists enzymatic reduction, accumulated as visible granules and was orders of magnitude more phototoxic.^63^ Neutral leuco-MB escapes mitochondrial trapping and redistributes freely, so the apparent diffuse distribution of MB is itself a consequence of its reduction.

Actual photodamage occurs where MB reduction is disfavored. In lysosomes, MB accumulates *via* endocytic uptake^64^ and retains its photoactive cationic form, contrasting with the mitochondrial trap where it is rapidly reduced to leuco-MB. Photosensitization then compromises vacuolar ATPase function and lysosomal integrity.^65^ At the plasma membrane, surface-bound MB generates ^1^O_2_ that can reach nearby lipids within the short diffusion radius of singlet oxygen (∼100–200 nm). In the cytoplasm, continuous re-oxidation of leuco-MB by O_2_ creates transient photodynamic windows.^66^ Parallel lysosomal and mitochondrial photodamage is synergistically lethal because autophagy-mediated recycling of damaged mitochondria requires intact lysosomes.^67^ In antimicrobial PDT, where MB is most successful clinically, the mitochondrial trap is absent entirely because bacteria lack mitochondria, and MB acts at the microbial surface.^68^ In cancer PDT, lysosomal damage appears to be the primary effector.^69^

MB is also not purely a Type II/PS. Above ∼1 µM it dimerizes (*K*_eq_ ∼3.8 × 10^3^ M^−1^), shifting almost entirely to electron-transfer pathways.^70^ DNA binding, protein association, and hypoxia further shift the balance toward Type I electron-transfer pathways. With redox regeneration by cellular reductants (*e.g.*, NADH photooxidation), MB functions as a PC. Its reduction by Complex I is neither photobleaching nor photocatalysis but dark enzymatic inactivation through a non-productive pathway.

#### 3.1.2 CV remains active at mitochondria

CV also accumulates in mitochondria as a lipophilic cation, but unlike MB it undergoes irreversible one-electron reduction to an unstable carbon-centered radical, rather than clean two-electron reduction to a stable leuco form (Fig. 3c).^71–73^ This radical autooxidizes to generate superoxide and hydrogen peroxide (H_2_O_2_). CV therefore retains its chromophoric form at the target organelle while simultaneously generating toxic radical intermediates.

Controlled experiments provided unequivocal evidence that light enhances the cytotoxicity of CV when it is bound to macromolecules or membranes. In free solution, the three dimethylaminophenyl rings of CV undergo ultrafast torsional relaxation (∼1–5 ps), completely outcompeting intersystem crossing (ISC) and preventing any photochemistry.^74^ Binding to macromolecules or membranes restricts this relaxation. Bovine serum albumin (BSA) binding increases photobleaching 18-fold and extends fluorescence lifetime from ∼5 ps to ∼0.4/3.6 ns, indicating substantial ISC enhancement.^75^ The resulting photoreduction generates the same carbon-centered radical observed in dark reduction, producing O_2_˙^−^ and H_2_O_2_ through a Type I mechanism with no detectable ^1^O_2_.^72,76^ This is consistent with a PI role. Whether partial regeneration through radical autooxidation qualifies CV as a PCI remains unresolved.

### 3.2 From a paradox to a design principle

Traditional PDT relies on bulk generation of singlet oxygen in the cytoplasm, requiring micromolar PS concentrations and high light doses to overwhelm antioxidant defenses.^77^ The subcellular site of photodamage, and not total oxidant burden, determines the cell death pathway and efficiency.^55,78,79^ Organelle-targeted photosensitizers can achieve effective killing at nanomolar concentrations, up to three orders of magnitude below conventional protocols.^7,58^

When embedded in an organelle membrane, a photosensitizer may shift from generating diffusible ^1^O_2_ (Type II/PS) to direct electron-transfer oxidation of lipids (Type I). If this initiates a self-propagating lipid peroxidation chain, the absorber acts as a PI or PCI and, consequently, one photochemical event produces many oxidized lipids through chain amplification. The truncated lipid products, not the initial hydroperoxides, permeabilize membranes and trigger cell death (Section 4.3). This shift from stoichiometric singlet oxygen chemistry to chain-amplified radical chemistry explains why membrane affinity and Type I competence predict cell killing better than *Φ*_Δ_.

The MB *versus* CV comparison maps directly onto this model (Fig. 3a). MB operates as a Type II/PS at lysosomes and the plasma membrane, and can function as a contact-dependent^80^ Type I/PC when regenerated through a redox cycle. Its dominant intracellular fate, however, is dark reduction to leuco-MB by Complex I, a non-productive pathway that is neither photobleaching nor photocatalysis. CV, when conformationally restricted at mitochondrial membranes, acts as a PI generating radical that can propagate lipid peroxidation at cardiolipin-rich membranes.

For PDT, next-generation photosensitizers should therefore prioritize organelle-specific membrane embedding and Type I competence over singlet oxygen yield. This argument is reinforced in the hypoxic environment of solid tumors, where pO_2_ is typically 5–30 mmHg (*vs.* 30–50 mmHg in normal tissue and ∼160 mmHg in air) and the concentration of O_2_ falls to ∼10–40 µM,^81^ kinetically suppressing Type II energy transfer and favoring contact-dependent Type I chemistry. PDT-driven O_2_ depletion within seconds of irradiation has further motivated the fractionated light schemes that have improved clinical efficacy.^81^ For suncare, the same principle implies that endogenous photosensitizers embedded in mitochondrial or lysosomal membranes, however modest their *Φ*_Δ_, can initiate chain-amplified damage that overwhelms antioxidant defenses (Sections 7 and 8).

## 4 Membrane photodamage: from lipid oxidation to pore formation

Across structurally diverse photosensitizer classes, a strong correlation exists between membrane binding efficiency and photocytotoxicity.^82–85^ This correlation is particularly striking when photosensitizers that produce comparable amounts of singlet oxygen are compared, because modifications that increase membrane affinity (zinc coordination and extension of an alkyl chain from methyl to octyl) dramatically enhance cell killing without changing photochemical yield,^86^ and phototoxicity tracks with the partition coefficient (log *P*) across a series of amphiphilic compounds.^84,85^

This observation presents an apparent paradox. Singlet oxygen is a diffusible species that can travel freely between the aqueous bulk and the lipid bilayer during its short lifetime,^87^ and quantitative measurements on giant unilamellar vesicles (GUVs) show that only one out of 270 ^1^O_2_ molecules reaching the membrane (fewer than 0.4%) actually reacts with a lipid double bond.^88^ If ^1^O_2_ were the sole mediator of membrane damage, physical attachment of the PS to the membrane should be unnecessary, and simply producing more ^1^O_2_ should suffice. Yet the experimental evidence overwhelmingly shows the opposite, despite the fact that the O_2_ concentration in lipid membranes is approximately one order of magnitude higher than in water and the lifetime of ^1^O_2_ is longer in membranes (>7 µs) than in water (∼3 µs).^87^ Triplet states of porphyrinoid PSs decay on a comparable microsecond scale, with bimolecular quenching by O_2_ approaching the diffusion limit (*k*_q_ ≈ 1–2 × 10^9^ M^−1^ s^−1^),^89^ so the productive step is set by local oxygen concentration and by the absorber's residence time at the lipid interface. Resolving this paradox required investigating the molecular-level oxidative processes that actually lead to membrane leakage.

### 4.1 Lipid oxidation pathways and their functional classification

The *cis*-unsaturated acyl chains of membrane phospholipids are the primary targets of photoinduced oxidative processes.^90,91^ Singlet oxygen, produced by Type II energy transfer, is an electrophile.^15,92^ It adds to lipid double bonds through the concerted ene reaction, in which an allylic hydrogen migrates as oxygen inserts, yielding allylic hydroperoxides with concomitant double-bond migration and an exclusive *trans* (*E*) configuration.^93,94^ This stereo- and regioselectivity distinguishes the ene pathway from radical-mediated oxidation, which produces both *E* and *Z* isomers at multiple chain positions.^95^ Because the ene reaction generates no radicals, each ^1^O_2_ molecule is consumed in a single stoichiometric oxidation event, and the absorber, having transferred its energy to O_2_ and returned to the ground state without chemical alteration, is fully regenerated. This is the hallmark of a Type II/PS. The maximum number of hydroperoxide groups per acyl chain equals its number of double bonds, so oleic acid (18 : 1) yields only a monohydroperoxide immune to further ^1^O_2_ attack,^96^ while linoleic acid (18 : 2) acid can form dihydroperoxides.^97^

When instead a membrane-embedded absorber in the triplet state directly abstracts a hydrogen atom or electron from a lipid substrate, the reaction enters a different regime. The most kinetically labile sites in polyunsaturated acyl chains are the bisallylic C–H bonds, whose bond dissociation enthalpies are approximately 30 kJ mol^−1^ lower than those of monoallylic C–H bonds, making them the preferential abstraction targets.^95^ Abstraction at a bisallylic position generates a pentadienyl radical (L˙) stabilized by delocalization across five carbons. Rapid trapping by O_2_ produces a lipid peroxyl radical (LOO˙) that propagates the chain by abstracting a hydrogen from a neighboring lipid, yielding a new hydroperoxide and a new L˙.^98,99^ Each propagation cycle consumes one lipid molecule, and the chain can run through tens to hundreds of cycles before termination. The accumulated hydroperoxides and their alkoxyl radical decomposition products undergo β-scission, cleaving the acyl chain to produce truncated lipid aldehydes, carboxylates, and ketones with no equivalent in the singlet oxygen pathway.

### 4.2 Hydroperoxides alter the structure but do not permeabilize

Lipid hydroperoxide is a primary product of both Type I and Type II photooxidation. In a lipid bilayer, the hydroperoxide group attached to an unsaturated alkyl chain interacts *via* hydrogen bonding with water molecules, bending the oxidized chain toward the aqueous interface. This results in a 15–20% increase in the lipid molecular area, provoking a decrease in bilayer bending rigidity, increased swelling between bilayers, and altered microviscosity near the hydroperoxide group. When GUVs containing unsaturated lipids are subjected to Type II photosensitized reactions, membrane fluctuations appear rapidly with subsequent release of buds, as the membrane equilibrates the area increase upon lipid hydroperoxide formation.^100,101^ Experiments using sodium azide (a ^1^O_2_ quencher) or GUVs composed exclusively of saturated lipids (*e.g.*, DPPC) showed no area increase upon irradiation, confirming that oxidation of unsaturated lipids by ^1^O_2_ is responsible for these perturbations.^88,102^ Despite these structural changes, no permeabilization was observed in phase contrast microscopy experiments when only lipid hydroperoxide was formed.^88,103^ MD simulations of POPC membranes confirmed that the Gibbs energy for water permeation does not change significantly when hydroperoxide or alcohol functional groups are added to the lipid.^80^ The presence of POPCOOH in the membrane can, however, have a synergistic effect on permeabilization promoted by electroporation.^104,105^

Quantitative GUV measurements have established distinct permeability regimes as a function of oxidized lipid fraction, from baseline low permeability at 0% oxidation, through strongly increased permeability at modest oxidation levels (a few to ∼10% oxidized lipids), to physical bilayer disruption with pore defects above approximately 12.5% oxidized lipid content.^106^ These two regimes (gradual permeability increase and catastrophic poration) involve distinct mechanisms, supporting the view that membrane photodamage is a continuum rather than a binary event. The presence of POPCOOH in membranes containing cholesterol and DPPC can induce liquid ordered to liquid disordered phase separation, resembling lipid rafts.^107^ Direct imaging of peroxidized lipid membranes has confirmed the emergence of phase separation in real time, with oxidized domains nucleating and coalescing on the scale of minutes.^108^ Notably, oxidation of PUFAs can attach more than one hydroperoxide group per chain, limited by the number of unsaturation sites. The impact of such polyhydroperoxides on the membrane structure remains largely unknown and deserves both experimental and theoretical investigation, given that PUFAs are important components of cell membranes, particularly in mitochondrial cardiolipin (Section 5.1).

### 4.3 Truncated lipids as the permeabilization trigger

In Type I reactions, lipid peroxides can be converted to alkoxyl radicals that form lipid aldehydes *via* β-scission.^80^ This cleavage generates shortened lipid structures, and several lines of evidence identify these truncated products as the molecular species responsible for membrane permeabilization. In MD simulations of POPC membranes, the Gibbs free energy for water permeation fell from 30 kJ mol^−1^ to 12 kJ mol^−1^ upon addition of lipid aldehydes.^80^ The simulations further showed that aldehyde lipids, by virtue of their high lateral mobility and truncated hydrophobic tails, diffuse and aggregate within the membrane, creating regions where their polar termini pull water into the bilayer interior.^109^ The high mobility of lipid aldehydes also allows interaction between lipids in opposing leaflets.

The unique permeabilizing capacity of truncated lipids has a structural basis. Native phospholipids with two long acyl chains adopt a cylindrical geometry favoring flat bilayer packing. Truncated oxidized lipids, with one intact chain and a short polar residual chain, adopt a conical geometry resembling single-tailed amphiphiles.^44^ This cone shape is energetically favorable at pore rims, where headgroup area must exceed tail volume.^108,110,111^ By partitioning preferentially to pore edges, truncated lipids reduce the line tension that normally drives rapid pore closure. Large pores in GUVs normally reseal within seconds, but cone-shaped amphiphiles can reduce line tension by up to two orders of magnitude and extend pore lifetimes from seconds to minutes.^112^

Pore formation follows predictable kinetics, accelerating with photosensitizer concentration, degree of lipid unsaturation, and light power, all of which increase the rate of truncated lipid production. For liposomes prepared with egg phosphatidylcholine, the presence of 15% POVPC (a lipid aldehyde) greatly decreased the initial time for pore opening, while increasing POVPC content to 30% increased water flux through the membrane under osmotic pressure differences.^44^ The rate of permeabilization increases not only with a higher number of double bonds per lipid chain but also when the unsaturation is closer to the polar head. A complete absence of oxidation was observed for vesicles with *trans* fatty acid chains, reinforcing the importance of *cis*-unsaturated lipid composition for membrane permeabilization. The localization of the photosensitizer is also essential for the kinetics of pore formation in a membrane, as discussed in Section 4.4.

Some evidence suggests that lipid peroxidation leads first to the formation of hydrophobic pre-pores permeable to amphiphilic molecules but not to ions. Upon further oxidation, these pre-pores can evolve to larger, non-specific ones. When photosensitization of GUV bilayers occurs predominantly at the outer leaflet, a monolayer area decrease (due to chain cleavage) leads to inner budding, while the opposite occurs for inner-leaflet oxidation.^113^ Membrane budding in such cases increases vesicle tension up to the lysis tension, creating pores with estimated diameters of 16–43 nm.

Cholesterol (20–30 mol% in the plasma membrane) reduces photoinduced permeabilization by scavenging singlet oxygen and peroxyl radicals, increasing membrane cohesion and condensing lipid packing, and reducing photosensitizer incorporation into the bilayer.^114^ MD simulations have shown that cholesterol specifically protects against water penetration in oxidized bilayers by maintaining hydrophobic core integrity even when neighboring lipids are oxidized.^115^ Cryo-EM structures of membrane pores have revealed cholesterol clusters at the protein–lipid interface, consistent with a structural role in stabilizing or modulating pore architecture.^116^ While cholesterol itself forms oxidation products that vary with reaction type,^117^ lipid acyl chain peroxidation remains the dominant driver of membrane damage. The cholesterol-poor inner mitochondrial membrane is therefore inherently more vulnerable to photoinduced permeabilization than the cholesterol-rich plasma membrane (Section 5.1).

### 4.4 Contact-dependent reactions: the paradigm shift

The finding that membrane permeabilization requires truncated lipid products reveals why membrane association so strongly influences photosensitizer biological activity. These aldehydes arise primarily from Type I reactions requiring direct contact between the excited absorber and lipid substrate.

**Fig. 4:**
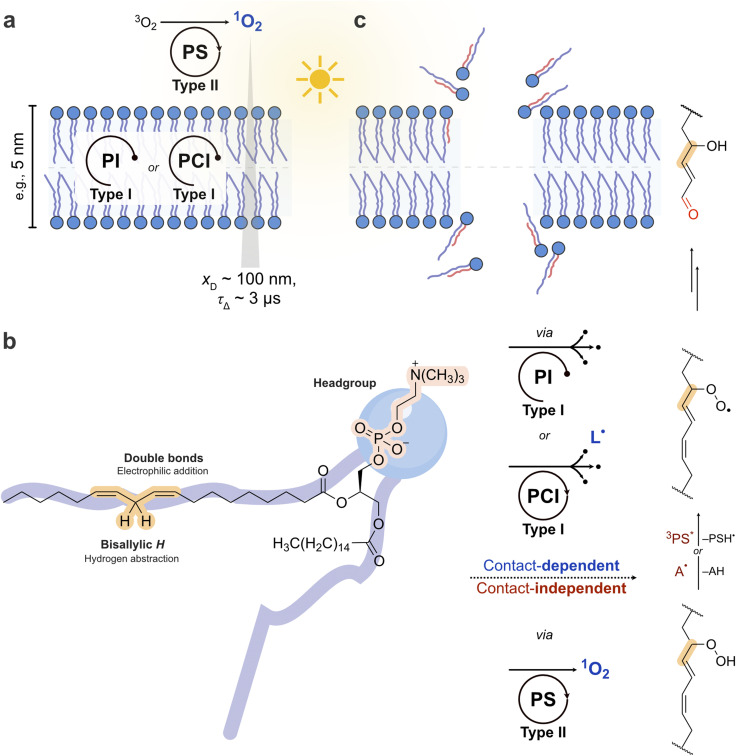
Molecular mechanism of membrane photodamage. (a) Two photosensitization pathways at a lipid bilayer. In the contact-independent pathway (top), an aqueous-phase absorber acting as a Type II/PS generates singlet oxygen that diffuses to the membrane (*x*_D_ is the distance and *τ*_Δ_ is the lifetime)^87,103,120,121^ and forms lipid hydroperoxides *via* the ene reaction.^93,94^ In the contact-dependent pathway (bottom), a membrane-embedded absorber acting as a PI or PCI abstracts a hydrogen atom from an unsaturated lipid (Type I), generating a carbon-centered radical (L˙) that enters a self-propagating peroxidation chain. (b) Molecular targets on a representative phospholipid. Singlet oxygen undergoes electrophilic addition across double bonds, whereas the excited absorber abstracts a bisallylic hydrogen. (c) Membrane-level consequences. Hydroperoxides from the ene reaction reorient their polar heads toward the aqueous interface, increasing membrane area (∼15–20%)^100,101^ and inducing budding without permeabilization.^88,103^ β-Scission of chain-propagated lipid peroxides produces truncated aldehydes that aggregate and draw water into the bilayer interior, forming transmembrane pores (estimated diameter 16–43 nm).^80,98,113^ Membrane permeabilization correlates with absorber membrane affinity and Type I engagement, and not with singlet oxygen quantum yield.^44,109,118^

The distinction between contact-independent and contact-dependent membrane damage maps onto the functional roles defined in Section 2 (Fig. 4). Contact-independent damage proceeds primarily through Type II photosensitization, in which the absorber transfers energy to O_2_, singlet oxygen diffuses to the membrane, and hydroperoxides accumulate without pore formation. The absorber functions as a Type II/PS. Contact-dependent damage proceeds through Type I mechanisms where the membrane-embedded absorber directly abstracts electrons or hydrogen atoms from lipids. When the resulting lipid radical is intercepted by a chain-breaking antioxidant before propagating, the absorber functions as a PC, producing individual oxidation products without chain amplification. When instead the lipid radical propagates a self-sustaining peroxidation chain culminating in truncated lipids and pore formation, the absorber acts as a PI (if consumed) or a PCI (if regenerated through a redox cycle). The vastly greater efficiency of membrane-embedded photosensitizers in producing truncated lipids and pores thus reflects a mechanistic shift from stoichiometric oxidation (Type II/PS or PC) to chain-amplified peroxidation (PI or PCI). This distinction explains why singlet oxygen quantum yield is a poor predictor of membrane damage and cell killing, while membrane affinity and Type I engagement are far superior predictors.

Our group demonstrated this principle by comparing the phenothiazinium photosensitizer DO15 with methylene blue under conditions designed to separate contact-dependent from contact-independent photochemistry.^80^ DO15 exhibited 27-fold higher colocalization with ω-9 lipid double bonds than MB, resulting in approximately one order of magnitude higher membrane permeabilization efficiency. A much lower production of lipid alcohols and lipid aldehydes was observed for MB, confirming the importance of contact-dependent reactions for aldehyde formation and pore opening. Crucially, the differential permeabilization could not be explained by differences in ^1^O_2_ production. Similar results were observed across a series of phenothiazinium compounds positioned inside the lipid bilayer, among which the one with the highest tendency to receive and stabilize an electron from unsaturated lipids permeabilized membranes most efficiently, despite producing similar or even lower ^1^O_2_ than the others.^118^ The importance of both membrane colocalization and electron-transfer reactions was further demonstrated with two pterins of different lipophilicity.^118,119^ When excited with UV light, both pterins can act by Type I and Type II mechanisms; however, only the more lipophilic pterin, which was buried deeply in the membrane, showed significant production of truncated lipids and higher membrane permeabilization. This compound also showed higher phototoxicity in HeLa cells, demonstrating a clear correlation between contact-dependent membrane damage and cellular toxicity.

### 4.5 Open questions in membrane photobiology

Research over the last three decades has considerably advanced our understanding of the mechanisms of photosensitized lipid oxidation and their consequences for the membrane structure, but several questions remain. Although the role of lipid monohydroperoxides in membrane damage has been addressed, little is known about polyhydroperoxides produced by oxidation of PUFAs. The importance of lipid hydrolysis during photosensitization of membranes has also been underexplored. Lysolipids and free fatty acids are products of lipid hydrolysis known to perturb membrane stability, morphology, and permeability. The kinetics of carboxylic ester hydrolysis are normally slow, but can be accelerated by interaction of cationic amphiphilic drugs with lipids. Some photosensitizers in the excited state may, with proper colocalization in the bilayer, catalyze lipid hydrolysis, contributing to membrane disruption. Lipid oxidation can also affect hydrolysis kinetics, and the correlation between these processes needs better understanding. Finally, membrane permeabilization can be exploited not only to damage organelles or the plasma membrane but also to internalize therapeutic macromolecules *via* endosome membrane disruption. This procedure, known as photochemical internalization, has been found to have potential for application in cancer treatment.

## 5 Organelle-specific photodamage: why the target membrane determines cell fate

Membrane permeabilization sets in motion regulated cell death programs that differ in initiating biochemistry, in the membrane that is breached, and in whether the dying cell is recognized by the immune system.^122^ Apoptosis is driven by caspase-mediated proteolysis downstream of mitochondrial outer membrane permeabilization (intrinsic pathway) or death-receptor engagement (extrinsic pathway), and is generally immunologically silent. Necroptosis depends on RIPK3/MLKL-mediated plasma-membrane rupture when caspase-8 activity is compromised. Pyroptosis is executed by gasdermin family pore formation (gasdermin D or E; GSDMD or GSDME) downstream of inflammasome assembly or caspase-3 cleavage, releasing IL-1β and IL-18. Ferroptosis is the iron-dependent cell death driven by uncontrolled lipid peroxidation at organelle membranes (Section 5.3). The latter three modalities are immunogenic, releasing damage-associated molecular patterns (DAMPs) that prime systemic anti-tumor responses, while apoptosis tends to dampen them.^123^ Effective PDT does not select a single modality. The most successful regimens permeabilize multiple organelle membranes and engage these pathways in parallel.

Membrane permeabilization by truncated lipids can damage any organelle. What determines whether a cell survives, dies by apoptosis, or dies by ferroptosis is which membrane is breached, because each compartment stores a different set of lethal effectors behind its membrane. This section examines how photodamage to mitochondria, lysosomes, and combinations thereof activates distinct regulated cell death pathways, and why these outcomes cannot be predicted from photosensitizer photochemistry alone. The relationship between subcellular photodamage and cell fate decisions has been extensively reviewed,^7,124^ and here we focus on the mechanistic principles that connect membrane-level events to downstream signaling.

### 5.1 Mitochondrial photodamage

Mitochondria sit at the intersection of energy metabolism and death signaling, making them uniquely potent targets for photodamage.^125^ The inner mitochondrial membrane (IMM) is especially vulnerable to photoinduced oxidation for three reasons: (i) it is cholesterol-poor, maximizing lipid-chain accessibility, (ii) it is enriched in polyunsaturated fatty acids, providing abundant oxidizable substrates, and (iii) it contains 15–20% cardiolipin (CL) by mass, a tetra-acyl diphosphatidylglycerol found almost exclusively in mitochondria.^126^

The four acyl chains of cardiolipin (typically all unsaturated) and comparatively small headgroup create a cone-shaped molecule that promotes the non-bilayer (hexagonal II) phase tendency responsible for the negative curvature stabilizing mitochondrial cristae.^111^ CL is essential for respiratory supercomplex organization and directly anchors cytochrome c to the IMM surface through electrostatic and hydrophobic interactions.^127^ The interplay between CL, other mitochondrial lipids, and their oxidation products in regulating membrane organization and apoptotic signaling has been reviewed.^128,129^ Photosensitizer-mediated oxidation of CL disrupts all of these functions simultaneously. As cristae remodel, electron transport chain efficiency drops, and the interaction anchoring cytochrome c to the IMM is weakened. This culminates in cytochrome c release into the cytosol, where it activates the apoptosome and initiates the caspase cascade.^129–133^

CL peroxidation can be initiated by Type II/PS (singlet oxygen ene reaction with polyunsaturated chains of CL) or by contact-dependent Type I reactions when the absorber is embedded in the IMM. In the Type I case, hydrogen atom abstraction from the bisallylic positions of CL generates CL radicals that enter the propagating chain of lipid peroxidation. Because CL carries four polyunsaturated chains and CL molecules cluster in cristae domains, the chain reaction amplifies damage well beyond the stoichiometric capacity of the original absorber. If the absorber operates in a closed redox cycle while initiating these chains, it functions as a PCI at the most vulnerable membrane in the cell.

**Fig. 5:**
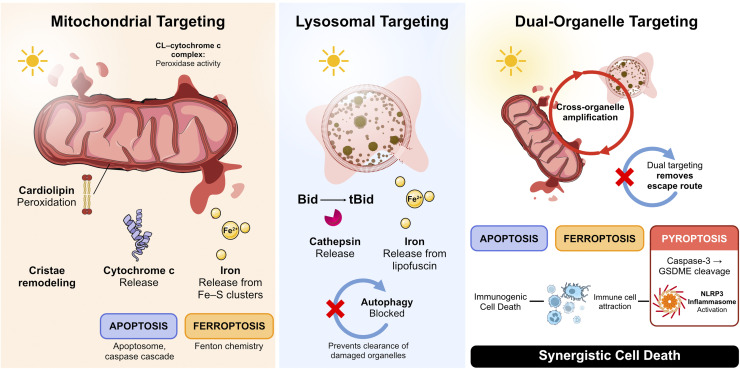
Organelle-specific photodamage and regulated cell death pathways. (Left) Mitochondrial targeting: an absorber at the inner mitochondrial membrane initiates cardiolipin (CL) peroxidation, either directly through singlet oxygen (Type II/PS) or through radical chain propagation along neighboring polyunsaturated CL acyl chains (PI/PCI). In both cases, partially oxidized CL triggers a biological amplification cascade in which cytochrome c acquires CL-specific peroxidase activity, expanding the pool of oxidized CL beyond the absorber's stoichiometric capacity. Consequences include cristae remodeling, cytochrome c release (apoptosis *via* apoptosome and caspase cascade), and iron liberation from Fe–S clusters (ferroptosis *via* Fenton chemistry). (Center) Lysosomal targeting: lysosomal membrane permeabilization releases cathepsins, which amplify mitochondrial damage *via* Bid cleavage and impair autophagic flux, preventing clearance of damaged organelles and producing irreversible cell death. (Right) Dual-organelle targeting engages apoptosis and ferroptosis, and can trigger pyroptosis (through caspase-3/GSDME cleavage or NLRP3 inflammasome activation) while eliminating autophagic rescue capacity. Release of IL-1β and IL-18 amplifies inflammation that synergizes with the canonical immunogenic cell death signature (ER stress-driven calreticulin exposure, HMGB1 release, and ATP secretion) to prime systemic anti-tumor immunity. Cross-organelle amplification cascades explain why dual targeting achieves potency far exceeding that of either pathway alone.

A biological amplification mechanism operates downstream of the initial photochemical event. When CL is partially oxidized, the CL–cytochrome c complex undergoes a conformational change in which cytochrome c acquires cardiolipin-specific peroxidase activity.^126^ In this altered conformation, cytochrome c catalyzes selective peroxidation of CL using H_2_O_2_ as the oxidant, generating a rapidly expanding pool of oxidized CL far exceeding what the original absorber could produce. This is a dark enzymatic process that does not require light, meaning that a relatively small photochemical initiation event can trigger a disproportionately large oxidative cascade. The absorber functions as a PI or PCI for the initial oxidation (Fig. 5).

This multilayered amplification (photochemical initiation followed by enzymatic CL peroxidation, cristae remodeling, supercomplex disruption, and further H_2_O_2_ generation in a feed-forward loop) explains why even nanomolar concentrations of mitochondria-targeted photosensitizers can trigger apoptosis, while micromolar concentrations of broadly distributed compounds acting through diffusible ^1^O_2_ fail to do so. The cationic photosensitizer CisDiMPyP, driven to mitochondria by the negative membrane potential, kills tumor cells through this cardiolipin–cytochrome c amplification cascade. Notably, the structurally related but anionic TPPS_2a_, which accumulates in lysosomes *via* endocytosis, achieves IC_50_ values in the low nanomolar range (∼30 nM), an order of magnitude below CisDiMPyP, but does so through the autophagy-associated lysosomal pathway discussed in Section 5.2 rather than through the mitochondrial cascade above.^134^ Across a series of phenothiazinium dyes, mitochondrial localization proved the strongest predictor of phototoxicity, outperforming singlet oxygen quantum yield as an indicator of cell death.^135^

### 5.2 Lysosomal photodamage

While mitochondrial targeting triggers rapid apoptotic death, lysosomal photodamage activates a distinct and often more effective killing strategy (Fig. 5). Lysosomes are terminal degradative organelles containing an arsenal of hydrolytic enzymes (cathepsins B, D, and L) active under acidic conditions.^136^ Lysosomal membrane permeabilization (LMP) releases these enzymes into the neutral cytosol, where they retain partial activity and cleave a broad spectrum of substrates. Cathepsin-mediated cleavage of pro-apoptotic Bcl-2 family proteins (*e.g.*, Bid cleavage to tBid) can amplify mitochondrial outer membrane permeabilization (MOMP), creating a cross-organelle amplification cascade.^122,137^

Critically, LMP also disrupts autophagy, the cell's primary mechanism for recycling damaged organelles.^138,139^ Autophagosomes that cannot fuse with functional lysosomes accumulate, and damaged mitochondria that would normally be cleared by mitophagy persist, generating sustained oxidant production and amplifying oxidative stress. This dual effect, direct cathepsin-mediated damage plus autophagy blockade, explains why lysosomal targeting can achieve more durable cell killing than mitochondrial targeting alone.^134^ Cells that survive initial mitochondrial photodamage can recover through mitochondrial biogenesis, but cells with compromised lysosomal function lose the ability to clear any subsequent organelle damage.

Lysosome-targeted iridium(iii) complexes achieved higher phototoxicity indices than mitochondria-targeted analogues, precisely because lysosomal damage blocked the autophagic rescue pathway.^140^ Photosensitizers localizing to lysosomes induced sustained lysosomal alkalinization, cathepsin release, and accumulation of non-functional autophagosomes, producing a delayed but irreversible cell death distinct from the rapid apoptosis triggered by mitochondrial PS.^141^ In short, lysosomal photodamage kills not by the initial insult but by eliminating the cell's capacity to recover from any subsequent one.

### 5.3 Dual-organelle and multi-compartment strategies

The most potent cell-killing strategies exploit the mechanistic crosstalk between mitochondrial and lysosomal damage. When both organelles are simultaneously compromised, the cell faces a lethal combination in which cytochrome c release activates apoptosis, cathepsin release degrades survival proteins, and autophagy blockade prevents recovery from either insult (Fig. 5).

Photosensitizers capable of damaging both mitochondria and lysosomes can engage multiple regulated cell death pathways.^64^ The ferroptotic component is significant, because mitochondria and lysosomes are both major intracellular iron reservoirs, storing iron in Fe–S clusters and cytochromes (mitochondria) and in ferritin undergoing autophagic degradation (lysosomes). When both compartments are breached, the combined iron release overwhelms the iron-chelating capacity of cells, feeding Fenton chemistry that generates hydroxyl radicals initiating additional lipid peroxidation chains in a feed-forward cycle.

Non-enzymatic lipid peroxidation initiated by photodynamic therapy bypasses the canonical ferroptosis pathway triggered by inhibition of glutathione peroxidase 4 (GPX4), the selenoenzyme that reduces phospholipid hydroperoxides within membranes. PDT-initiated peroxidation operates independently of GPX4 and can therefore kill cells resistant to conventional ferroptosis inducers.^142^ Live-cell imaging with fluorogenic radical-trapping probes has now enabled direct visualization of ferroptotic lipid peroxidation as it initiates and propagates through cellular membranes, confirming that the onset is spatially localized and spreads in a wave-like manner consistent with a chain-propagating mechanism.^143^

Emerging evidence also links photodynamic treatment to pyroptotic cell death. Caspase-3 activated by the apoptotic cascade can cleave gasdermin E, releasing its pore-forming *N*-terminal domain and converting apoptotic signaling into inflammatory cell death.^144^ NLRP3 inflammasome activation by PDT has also been reported, with Golgi apparatus photodamage identified as a key trigger that upregulates NLRP3 expression and promotes caspase-1-dependent GSDMD cleavage.^145^ Additional proposed activation signals include lysosomal membrane permeabilization with cathepsin B release and potassium efflux, although the direct mechanistic links between each organelle-specific lesion and inflammasome assembly remain incompletely characterized.^146^ The resulting release of IL-1β and IL-18 amplifies inflammation that, together with oxidant-mediated endoplasmic reticulum stress and the spatiotemporal release of DAMPs (calreticulin exposure, ATP secretion, and HMGB1 release), defines the canonical immunogenic cell death signature primed by PDT and capable of eliciting systemic anti-tumor responses.^145–147^

The principle emerging from these studies is that the most effective photodynamic strategies do not maximize ^1^O_2_ production but rather maximize the number of critical organelle membranes permeabilized per photon absorbed. Dual-organelle targeting achieves this by engaging multiple lethal pathways simultaneously while eliminating the cell's capacity for self-repair. The same organelle-targeting principles that govern PDT efficacy also explain why sunlight causes cumulative tissue damage, because endogenous photosensitizers concentrated in mitochondria and lysosomes engage identical membrane permeabilization mechanisms under chronic low-dose irradiation. The distinction between PDT and sun damage is one of dose rate and intent, not of mechanism.

## 6 Redox homeostasis: from eustress to distress

Photodamage is not binary. A cell hit by the same photosensitizer dose can mount a protective response or die, depending on its antioxidant reserves at the moment of irradiation. This section examines the redox equilibrium that sets that threshold. Every cell maintains a dynamic redox equilibrium that determines whether a given oxidative insult is absorbed, signaled, or lethal. Understanding this equilibrium is essential for interpreting both PDT efficacy and cutaneous photodamage, because the same photosensitizer dose can produce oxidative eustress (adaptive signaling) or distress (irreversible damage) depending on the cell's antioxidant capacity at the moment of irradiation.

### 6.1 Cellular redox landscape and antioxidant defense

Aerobic metabolism continuously generates superoxide, hydrogen peroxide, hydroxyl radicals, and, *via* nitrogen pathways, peroxynitrite. Mitochondria dominate intracellular production (0.1–0.2% of consumed O_2_), but in skin the exposome (solar radiation, pollution, and topical products) often contributes more than endogenous sources. Cells also produce oxidants deliberately. NAD(P)H oxidases generate O_2_˙^−^ for calcium-dependent signaling, and nitric oxide synthases produce NO˙ for vascular and immune regulation. These regulated sources constitute oxidative eustress, controlled oxidant production that activates adaptive signaling rather than causing damage. The boundary between eustress and distress is not defined by the identity of the reactive species but by its concentration, duration, and spatial distribution, a principle that directly parallels the localization-dependent outcomes observed in PDT. Just as a mitochondria-targeted PS at nanomolar concentration triggers specific apoptotic signaling while a broadly distributed PS at micromolar concentration causes nonspecific necrosis, endogenous oxidants at physiological concentrations activate responses mediated by nuclear factor erythroid 2-related factor 2 (Nrf2), while sustained overproduction overwhelms defenses and drives pathology. Whether the cell absorbs or succumbs to a given oxidative insult depends on the capacity of its antioxidant systems at the moment of challenge.

Cellular antioxidant defense rests on the thioredoxin–peroxiredoxin (Trx–Prdx) system, which detoxifies peroxides in a compartment-specific manner, and the glutathione (GSH)–glutaredoxin system, which maintains thiol homeostasis and supports glutathione peroxidase (GPx) activity. Superoxide dismutases (SOD1/SOD2) convert O_2_˙^−^ to H_2_O_2_, cleared downstream by catalase and the peroxidase systems. Both the Trx and GSH systems depend on NAD(P)H as the ultimate electron donor, supplied primarily by the pentose phosphate pathway (PPP). The PPP is rapidly activated in skin cells under oxidative stress, including UV exposure, to sustain redox balance. This metabolic coupling means that any process depleting NAD(P)H, including the photoredox catalysis of NADH described in Section 2,^49^ simultaneously compromises antioxidant defense capacity, creating a vulnerability that photosensitizers can exploit.

**Fig. 6:**
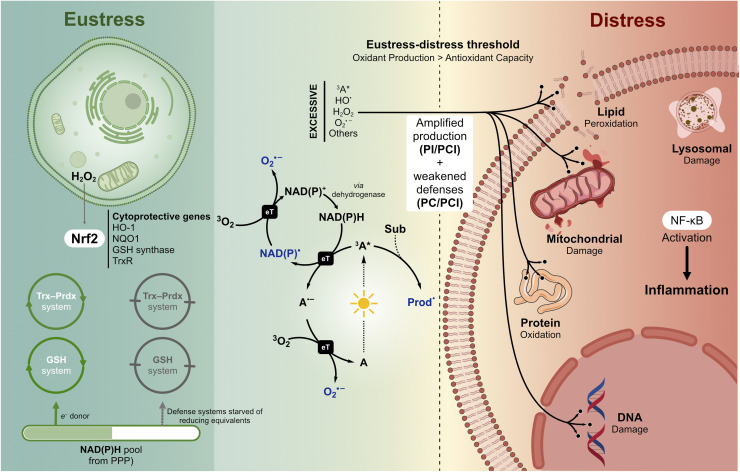
Photosensitization and the eustress-distress transition. At low oxidant flux (left), H_2_O_2_ activates Nrf2-mediated cytoprotective signaling, and NAD(P)H from the pentose phosphate pathway sustains the Trx–Prdx and GSH antioxidant systems (eustress). The central catalytic cycle shows how a single Type I turnover of an absorber (A) simultaneously generates superoxide and consumes NAD(P)H. The triplet-excited absorber (^3^A*) oxidizes NAD(P)H by using one electron, producing a semireduced absorber (A˙^−^) and NAD(P)˙ that may reduce oxygen to produce O_2_˙^−^.^148^ In this scenario, each turnover produces up to two superoxide equivalents while consuming one NAD(P)H. When downstream radicals initiate self-propagating lipid peroxidation chains (PI/PCI behavior), oxidant production is amplified beyond the stoichiometric capacity of the absorber. Concurrently, catalytic NAD(P)H consumption (PC/PCI behavior) starves the Trx–Prdx and GSH systems of reducing equivalents. This combination of amplified oxidant production and weakened antioxidant defense pushes the cell across the eustress-distress threshold into membrane peroxidation, NF-κB-driven inflammation, and organelle damage (right).

The vulnerability of NADPH-dependent defenses becomes apparent when the roles defined in Section 2 are mapped onto this redox landscape (Fig. 6). A chromophore acting as a PI generates radicals that initiate lipid peroxidation chains propagating independently of the original absorber, amplifying oxidant production beyond the stoichiometric capacity of the chromophore itself. A chromophore acting as a PC, with NADPH or NADH as the cycle-closing reductant, catalytically oxidizes the very reducing equivalents that sustain the Trx–Prdx and GSH defense systems. Each catalytic turnover simultaneously regenerates the absorber and consumes one molecule of NAD(P)H. The most damaging scenario arises when these roles converge in a single species. A chromophore operating as a PCI both amplifies oxidant production through downstream radical chains and depletes the defenses meant to contain them, because the same catalytic cycle that regenerates the absorber and produces chain-initiating superoxide also consumes NAD(P)H. This dual attack, amplified offense combined with weakened defense, is precisely what tips the cell from eustress into distress.

### 6.2 Skin-specific antioxidant mechanisms

The skin's antioxidant defenses include enzymatic systems (SOD, catalase, and Prdx) and non-enzymatic components (melanin, uric acid, vitamins C and E, carotenoids, coenzyme Q10, and α-lipoic acid), distributed in a gradient with the highest concentrations in the stratum corneum. These defenses are orchestrated by what has been termed the “redox code”, a regulatory framework that links specific oxidative modifications to defined signaling outcomes.^50,149^

The master regulator Nrf2, stabilized when oxidative stress disrupts its degradation by Kelch-like ECH-associated protein 1 (Keap1), upregulates cytoprotective genes encoding heme oxygenase-1 (HO-1), NAD(P)H quinone dehydrogenase 1 (NQO1), glutathione synthases, and thioredoxin reductase, enhancing resilience to subsequent oxidative challenges. This adaptive response is central to the concept of oxidative eustress in skin: moderate, transient oxidant production activates Nrf2 and strengthens defenses, while their chronic or excessive generation overwhelms the system.

Chronic oxidant production disrupts redox balance, leading to membrane peroxidation, impaired antioxidant defenses, and activation of pro-inflammatory transcription factors (AP-1 and NF-κB). This cascade exacerbates cutaneous disorders including photoaging, hyperpigmentation, and carcinogenesis, underscoring the need for robust antioxidant strategies in dermatology and photoprotection.

### 6.3 Bridging to visible light photodamage

Sunlight encompasses a broad spectrum of electromagnetic waves, including ultraviolet (UV-C, UV-B, and UV-A), visible light, and infrared radiation. While UV-A and UV-B radiation are well documented for their distinct damaging effects on the skin, visible light, particularly high-energy violet and blue light between 400 and 500 nm, has emerged as a significant yet understudied contributor to cutaneous photodamage. The preceding sections provide the tools to ask the right question: not how much total oxidant visible light generates, but which endogenous photosensitizers it activates, where they sit, and whether they initiate radical chains. The following section applies this analysis to the principal endogenous chromophores responsible for visible light photodamage.

## 7 Endogenous photosensitizers and skin photodamage

**Fig. 7:**
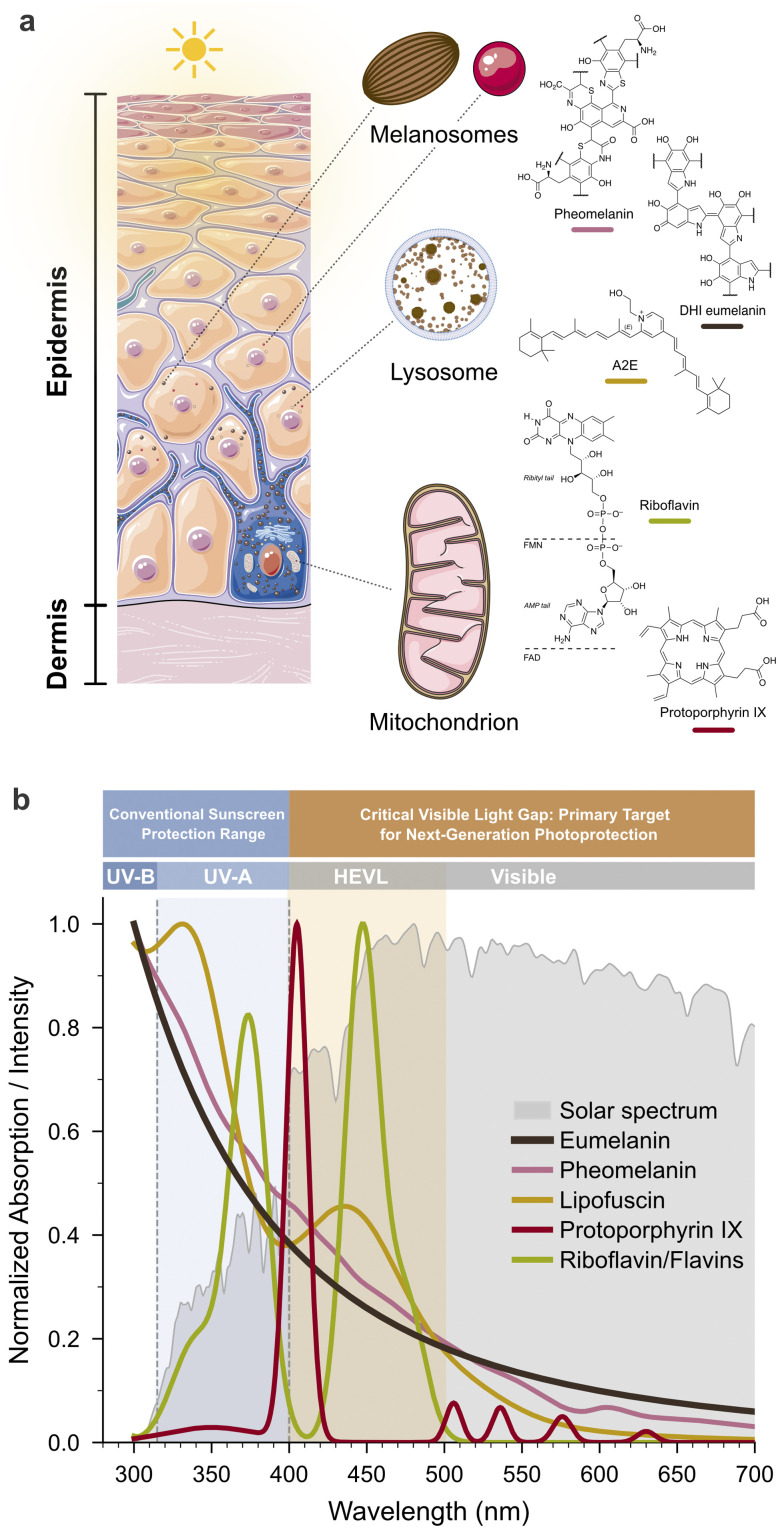
Endogenous chromophores in human skin. (a) Schematic cross-section of the epidermis and upper dermis showing the subcellular localization of principal endogenous chromophores. A dendritic melanocyte at the basal layer synthesizes and transfers melanosomes to surrounding keratinocytes. Callouts highlight the three organelle compartments relevant to endogenous photosensitization, with representative chemical structures shown alongside. Eumelanin (dark brown, elongated granules) dissipates >99.9% of absorbed photon energy as heat through ultrafast internal conversion and is not an efficient photosensitizer.^150^ Pheomelanin (reddish-brown granules) generates superoxide and H_2_O_2_, functioning as a PI/PCI. Lipofuscin (yellow-green lysosomal aggregates) generates radicals that initiate lipid peroxidation chains, operating as a PI/PCI. PpIX (mitochondria) acts as a Type II/PS, and riboflavin/flavoproteins (mitochondria) function as membrane-associated PI/PCI (**Box 1**). (b) Spectral overlap between solar radiation and endogenous skin chromophores. Normalized absorption spectra of eumelanin (power-law model),^151^ pheomelanin,^152^ protoporphyrin IX, riboflavin,^153^ and lipofuscin^154^ are overlaid on the ASTM G173-03 AM1.5 global tilt solar irradiance spectrum (gray fill). Each absorption curve is normalized to its peak value within the displayed range. Conventional broad-spectrum sunscreens primarily attenuate UV-B and UV-A radiation (blue region), leaving a gap in the visible range (amber region) where all four endogenous photosensitizers retain significant absorption.

The principles established through PDT research, that localization determines the outcome, that direct PS–lipid reactions drive membrane permeabilization, and that organelle-specific damage activates distinct death pathways, apply directly to understanding how sunlight damages skin. Human skin contains multiple endogenous chromophores that act as photosensitizers through distinct mechanisms (Fig. 7).^15^ This section examines the main examples through the mechanistic lens developed in Sections 2–6.

### 7.1 Lipofuscin: the aging photosensitizer

**Fig. 8:**
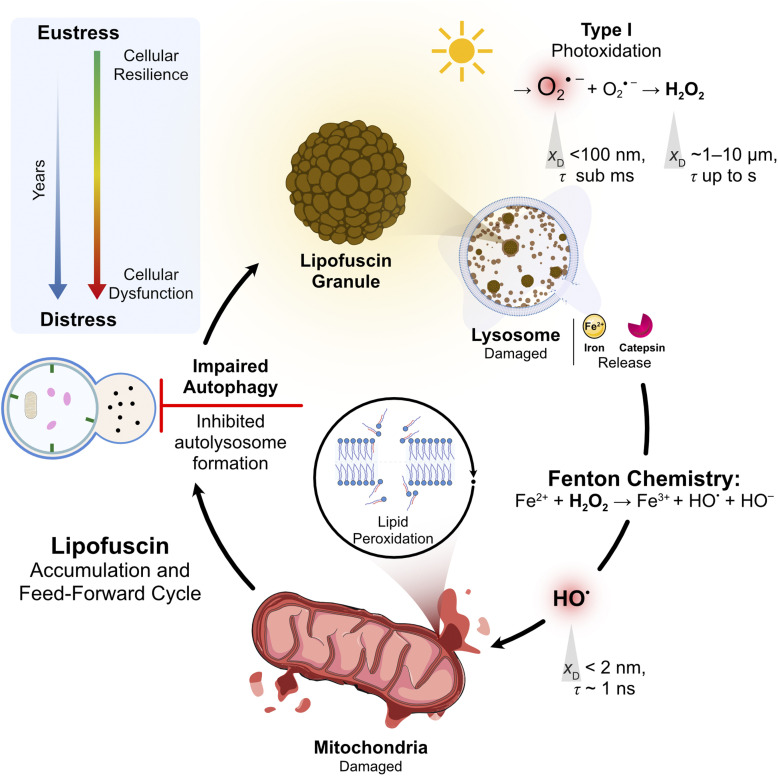
The lipofuscin feed-forward cycle. Lipofuscin absorbs visible light (400–500 nm) and generates superoxide and derived radicals through Type I reactions, functioning as a PI/PCI. These radicals damage both lysosomal membranes (releasing iron and cathepsins) and neighboring mitochondria. Labile iron feeds Fenton chemistry, amplifying lipid peroxidation chains independently of the original chromophore. The resulting oxidative damage impairs autophagy and lysosomal degradation capacity, leading to further lipofuscin accumulation and increased photosensitivity upon subsequent light exposure. Over years, this self-amplifying cycle progressively shifts the cell's resting redox state toward the eustress-distress threshold (Fig. 6), providing a molecular mechanism for the visible-light vulnerability of aging skin and a primary target for visible-light photoprotection (Section 8.1, Strategy 4). Diffusion distances (*x*_D_) and lifetimes (*τ*) were estimated from literature data.^159–162^

Lipofuscin is a complex, autofluorescent pigment, composed of cross-linked protein and lipid oxidation products, metal ions (particularly iron and copper), and organelle remnants that accumulates in postmitotic cells as autophagic and proteolytic clearance decline with age. Often called the “aging pigment,” its progressive accumulation reflects the interplay between oxidative stress and impaired lysosomal degradation (Fig. 8).^17,155–157^

Lipofuscin primarily accumulates in lysosomes, where it can interfere with normal cellular functions. Although it has sometimes been considered an inert waste product, lipofuscin is photochemically active: its heterogeneous chromophore composition produces broad absorption extending from the ultraviolet well into the visible range, with significant absorption between 400 and 500 nm. When exposed to light within this spectral window, lipofuscin generates superoxide and derived oxidants through predominantly Type I electron-transfer mechanisms, a behavior consistent with the photocatalytic initiator role, as detailed below, leading to oxidative damage within the cell. The accumulation of lipofuscin granules has been shown to increase the susceptibility of organelles, particularly mitochondria and lysosomes, to photoinduced damage.^158^ Also, lipofuscin is able to produce ^1^O_2_ in significant quantities, acting concurrently through Type I and Type II mechanisms.^17^

Critical quantitative evidence came from keratinocytes loaded with a lipofuscin-like material,^17^ which showed markedly increased sensitivity to UV-A and visible light irradiation compared to control cells. Elevated intracellular oxidant production, mitochondrial membrane potential dissipation, and lysosomal membrane permeabilization were observed at light doses innocuous to lipofuscin-free cells. Crucially, this photodamage established a feed-forward cycle: the initial oxidative insult impaired autophagy, reducing the cell's capacity to clear damaged organelles and oxidized aggregates, which in turn led to further lipofuscin accumulation and increased photosensitivity upon subsequent light exposure. This vicious cycle, photodamage leading to impaired clearance leading to more photosensitizer accumulation leading to greater photodamage, provides a molecular mechanism for the progressive increase in visible light vulnerability observed in aging skin.^18^

The feed-forward nature of lipofuscin photodamage also connects directly to the dual-organelle paradigm in Section 5. Lipofuscin resides in lysosomes but its photogenerated oxidants damage neighboring mitochondria. Lysosomal membrane permeabilization releases not only cathepsins but also the iron-rich lipofuscin itself into the cytosol, where labile iron feeds Fenton chemistry and amplifies lipid peroxidation in mitochondrial membranes. The result is simultaneous lysosomal and mitochondrial compromise, the same dual-organelle injury that produces the most effective cell killing in PDT, but occurring here under chronic low-dose visible light exposure during normal daily activities.

Lipofuscin is best understood as a photocatalytic initiator (PCI). Its heterogeneous chromophores absorb visible light and generate radicals *via* Type I electron-transfer reactions. These radicals initiate self-propagating lipid peroxidation chains that cause membrane damage independently of further photon absorption. The lipofuscin granule itself is consumed only slowly through gradual photobleaching, allowing sustained radical production over many catalytic cycles before significant chromophore loss occurs. This PCI behavior explains the disproportionate cellular damage observed from modest visible light doses in lipofuscin-laden cells: the damage is amplified not by the photochemical efficiency of any single chromophore within lipofuscin, but by the chain-propagating radical chemistry downstream of each photocatalytic turnover.

### 7.2 Melanin: paradoxical protector

Melanin is synthesized within melanosomes, lysosome-related organelles that reside in melanocytes, and transferred to surrounding keratinocytes, serving as the skin's primary photoprotective pigment.^163^ However, the two major forms of melanin, eumelanin and pheomelanin, have fundamentally different photochemical behaviors that map cleanly onto the PS/PC/PI/PCI classification.

Eumelanin, the brown-black pigment predominant in darker skin phototypes, is an extraordinarily efficient photoprotective absorber. It dissipates more than 99.9% of absorbed photon energy as heat through ultrafast internal conversion, with negligible triplet state population and minimal oxidant generation (see **Box 2** for the energy balance underlying this distinction).^150^ Eumelanin is not a photosensitizer in any functional sense: it absorbs light but does not induce physical or chemical changes in other molecular entities. It functions as a physical quencher, converting photon energy to thermal energy without generating excited states capable of activating substrates. This accounts for its well-established photoprotective role against UV-induced DNA damage and its correlation with reduced skin cancer risk.

Pheomelanin, the yellow-red pigment enriched in individuals with red hair and fair skin (phototypes I–II), presents a strikingly different photochemistry. Upon UV-A and visible light irradiation, pheomelanin generates O_2_˙^−^ and hydrogen peroxide through Type I electron-transfer reactions involving its benzothiazine and benzothiazole subunits.^164^ When superoxide-derived radicals initiate lipid peroxidation chains that propagate independently of further pheomelanin turnover, pheomelanin functions as a photoinitiator, a mechanistic distinction directly relevant to the elevated melanoma risk observed in individuals with the pheomelanin phenotype. Pheomelanin can also operate through Type II mechanisms, producing substantial amounts of ^1^O_2_ upon exposure to UV-A and visible light.^165^ Thus, although melanin pigments as a whole provide photoprotection through absorption and scattering, pheomelanin paradoxically acts as both a Type I and Type II photosensitizer, generating oxidative damage through multiple pathways.

A particularly striking manifestation of melanin photochemistry is the formation of cyclobutane pyrimidine dimers (CPDs) in melanocytes hours after UV exposure has ceased, a phenomenon termed “dark CPDs”.^166^ UV-excited melanin generates nitric oxide-derived species and superoxide, which combine to form peroxynitrite. Peroxynitrite then undergoes thermal decomposition to produce an excited-state carbonyl species (a dioxetane intermediate)^167^ with sufficient energy to transfer to DNA and induce CPD formation without any additional photon input. This chemiexcitation pathway means that melanin-derived reactive intermediates continue to cause DNA damage in the dark, extending the effective duration of UV mutagenesis well beyond the period of light exposure. Importantly, dark CPD formation was more pronounced in melanocytes containing pheomelanin than those containing only eumelanin, consistent with the greater photosensitizing activity of pheomelanin described above.^168^

The eumelanin–pheomelanin distinction has direct implications for the photoprotection strategy. Individuals with pheomelanin-dominant skin carry an endogenous Type I photosensitizer that generates oxidants under visible light, radiation that conventional sunscreens do not block. This population may benefit disproportionately from visible light photoprotection strategies and from antioxidant formulations designed to intercept the radical chains initiated by pheomelanin photochemistry.

Beyond its photochemical activity, melanin homeostasis depends on the proper turnover of melanosomes within keratinocytes. While functional melanosomes shield skin cells from photodamage, excessive or damaged melanosomes can accumulate when lysosomal function is compromised. Melanophagy, the selective autophagic clearance of melanosomes, plays a pivotal role in epidermal pigment homeostasis, and its impairment may contribute to dyspigmentation.^169^

**Box 2. Photothermal therapy (PTT) and the heat balance of PDT**

PTT and PDT sit at opposite ends of the same energy-balance spectrum. PTT operates through chromophores such as gold nanorods, polydopamine nanoparticles, and conjugated polymers, which funnel essentially all absorbed photon energy into ground-state vibrational modes *via* ultrafast internal conversion, increasing local tissue temperature; the productive species is heat, not an excited state.^170,171^ PDT is not athermal. At the porphyrin Soret band (∼3 eV per photon) and a ^1^O_2_ excitation energy of ∼1 eV, at least 2 eV per absorbed photon is dissipated as heat regardless of *Φ*_Δ_, so even an ideal Type II PDT agent converts no more than ∼33% of the absorbed photon energy into productive chemistry; with clinical *Φ*_Δ_ ≈ 0.5–0.6, the heat fraction approaches 80% per absorbed photon. At the irradiances used in clinical PDT (50–200 mW cm^−2^) the resulting tissue heating is normally a few degrees Celsius, well below the ∼43 °C (sustained for minutes)^172^ required for thermal damage. Above this threshold, the PDT and PTT regimes overlap and dosimetry must be designed accordingly.^173,174^ The same balance places eumelanin firmly on the PTT side of the contrast: more than 99.9% of absorbed photon energy is dissipated as heat (Section 7.2), the photophysical signature of a near-ideal physical quencher rather than that of a photosensitizer. Pheomelanin and lipofuscin, by contrast, divert a non-negligible fraction of the absorbed energy into excited-state photochemistry, which is the mechanistic basis of their phototoxicity (Section 7). An effective visible-light filter for skin must therefore replicate the photothermal dissipation of eumelanin, not the photosensitizing pathways of pheomelanin (Section 8.2).

### 7.3 Flavins, porphyrins, and other endogenous chromophores

In addition to lipofuscin and melanin, human skin contains several other endogenous chromophores with significant photosensitizing capacity. Their photochemistry, interpreted through the same lens, further reinforces the principle that localization and reaction type determine the biological outcome.

Riboflavin (vitamin B2) and its coenzyme derivatives FAD and FMN absorb strongly in the blue-violet region (absorption maximum at around 450 nm) and are present in all skin cell types. As discussed in Section 2, riboflavin is among the most mechanistically versatile endogenous photosensitizers: it operates concurrently as a Type II photosensitizer (generating singlet oxygen *via* energy transfer) and a Type I photocatalyst (oxidizing substrates *via* closed redox cycles). In the specific context of skin, riboflavin-mediated photooxidation of membrane lipids constitutes photocatalytic initiation (PCI), as described in Section 2.2: riboflavin abstracts hydrogen from unsaturated lipids, the riboflavin radical is reoxidized by O_2_, and the lipid radical enters a self-propagating peroxidation chain. In dermal fibroblasts, where flavin concentrations are substantial and mitochondrial flavoproteins are abundant, this mechanism contributes to visible light-induced oxidative damage that mirrors the mitochondrial photodamage produced by exogenous PS in PDT.

Protoporphyrin IX (PpIX), an intermediate in heme biosynthesis, accumulates in mitochondria and is a potent Type II photosensitizer (*Φ*_Δ_ ∼0.56; Soret band at 405 nm, Q bands 500–635 nm). Its clinical exploitation in ALA-PDT relies on overwhelming the heme pathway to accumulate PpIX selectively in rapidly dividing cells.^175^ However, basal levels of PpIX are present in all nucleated skin cells, and its mitochondrial localization means that even modest PpIX excitation by visible light can trigger the same cardiolipin oxidation and cytochrome c release pathway described in Section 5.1. PpIX thus represents an endogenous mitochondria-targeted photosensitizer whose activity under ambient visible light contributes to the baseline oxidative load on dermal and epidermal mitochondria.

NADH and NAD(P)H absorb in the UV-A range (absorption maximum at around 340 nm) and are present at high concentrations in metabolically active skin cells. While their direct photosensitizing activity is modest, their photochemical depletion has significant biological consequences. Exogenous photosensitizers can catalytically oxidize NADH to NAD^+^ through Type I photoredox cycles.^49^ Endogenous chromophores capable of similar photoredox activity, including flavins and porphyrins, may contribute to NADH/NAD(P)H depletion under prolonged light exposure. Because NAD(P)H is the ultimate electron donor for both the thioredoxin–peroxiredoxin and glutathione antioxidant systems (Section 6.1), its photocatalytic depletion simultaneously compromises the cell's capacity to neutralize the very oxidant generated by photosensitization. This convergence creates a synergistic vulnerability: photosensitizers generate oxidants while simultaneously depleting the reducing equivalents needed to detoxify them.

Additional endogenous chromophores contributing to cutaneous photodamage include advanced glycation end products (AGEs), which accumulate in dermal collagen with aging and absorb in the UV-A and blue light range, bilirubin, a heme degradation product present in skin at low concentrations, and tryptophan and its metabolites, which absorb in the UV-B and UV-A range and can generate superoxide and singlet oxygen upon excitation. The cumulative effect of these diverse endogenous chromophores, each with distinct absorption spectra, subcellular localizations, and photochemical mechanisms, is a complex photosensitization landscape in which visible light activates multiple parallel damage pathways simultaneously. Understanding this landscape through the mechanistic analysis above reveals why effective photoprotection must address far more than UV transmission.

## 8 Photoprotection beyond photon blocking

The preceding sections establish that photosensitizer localization, membrane contact, and organelle-specific damage determine the biological outcome far more than photochemical efficiency. These principles, derived from three decades of PDT research, have never been systematically applied to photoprotection. This section translates them into a rationale for next-generation suncare strategies that address the full spectrum of light-induced skin damage, including the visible light range that conventional sunscreens ignore.

### 8.1 Suncare strategies from mechanistic insights

**Fig. 9:**
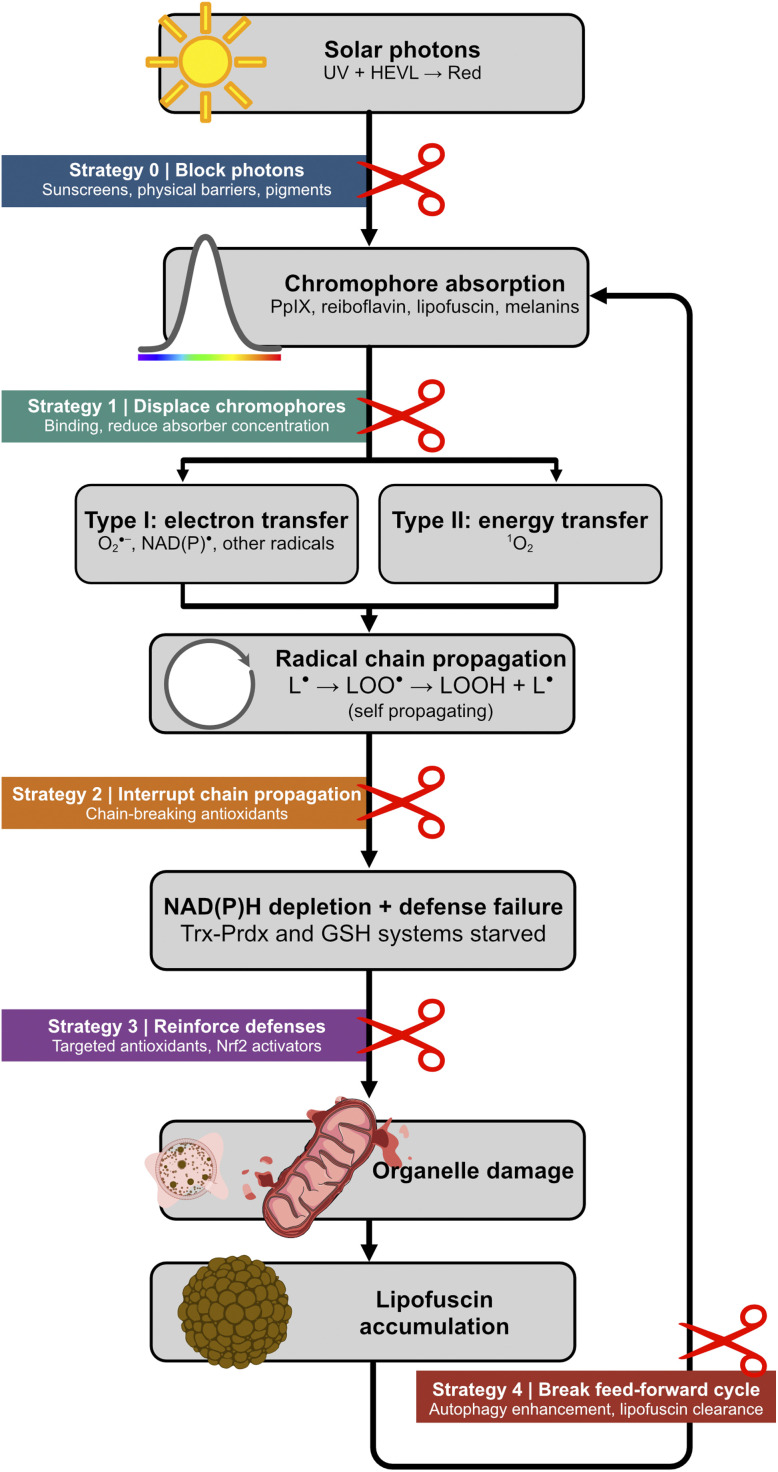
Photoprotection strategies derived from the PDT mechanistic analysis, mapped onto the photodamage cascade. Each strategy targets a distinct step: (0) photon blocking with UV filters and visible light absorbers (iron oxides and melanin-mimetic particles) for the 400–500 nm spectral gap; (1) competitive displacement of endogenous chromophores from critical membrane sites using non-photoactive amphiphilic molecules; (2) interruption of radical chain propagation with membrane-localized chain-breaking antioxidants (α-tocopherol and ubiquinol); (3) reinforcement of organelle-specific defenses through Nrf2 activation (sulforaphane and dimethyl fumarate) and mitochondria-targeted antioxidants (MitoQ and SkQ1); (4) breaking the lipofuscin feed-forward cycle *via* autophagy induction and iron chelation. The strategies are mechanistically complementary and are expected to provide greater-than-additive benefit when combined. The cascade integrates the mechanistic elements introduced in Fig. 4–8.

If the mechanisms of photodamage in PDT and sun exposure are fundamentally the same, differing only in whether the photosensitizer is exogenous or endogenous, and whether the goal is to maximize or minimize cell killing, then PDT research offers a roadmap for photoprotection that goes far beyond blocking photons at the skin surface. Four distinct strategies emerge from the mechanistic insights in Sections 3–7. The starting point is photon blocking itself (Strategy 0). Conventional UV filters address UV-B and UV-A, but the spectral gap in the 400–500 nm range demands extension to visible light absorbers such as iron oxides and melanin-mimetic particles (Section 8.2). The strategies below address the steps downstream of photon absorption (Fig. 9).

Strategy 1: prevent photosensitizer accumulation at sensitive subcellular sites. Since localization at critical membranes amplifies damage by orders of magnitude (Section 3), non-photoactive amphiphilic molecules competing with endogenous PS for mitochondrial or lysosomal membrane binding sites could redistribute oxidant production away from the most damage-sensitive locations without reducing total oxidant output. Direct demonstrations of this approach in human skin are not yet available, and we present Strategy 1 as a mechanistic hypothesis rather than a validated intervention. The principle of competitive displacement at hydrophobic binding sites does, however, have biophysical precedent: site-selective spectroscopic probes have been used to quantify competition between photosensitizers and structurally similar non-photoactive amphiphiles at the hydrophobic binding pockets of human serum albumin (*e.g.*, displacement of dansyl-l-proline by chlorin p6 at Sudlow site II).^176^ Whether analogous competition can be engineered to displace endogenous photosensitizers, particularly lipofuscin and flavin-binding proteins, from organelle membranes in human skin is, to our knowledge, untested, and we identify it as an explicit goal for the next generation of suncare actives.

Strategy 2: interrupt contact-dependent reactions with membrane-localized antioxidants. The contact-dependent paradigm (Section 4) establishes that truncated lipids produced by Type I reactions at the membrane are the proximate cause of pore formation and organelle permeabilization. This finding has direct implications for the antioxidant strategy. If endogenous photosensitizers function as photocatalytic initiators rather than simple Type II photosensitizers, then the dominant damage pathway is radical chain propagation in membranes, not singlet oxygen diffusion from the aqueous phase. Chain-breaking antioxidants that intercept lipid peroxyl radicals within the membrane, such as α-tocopherol (vitamin E) and coenzyme Q10 in its reduced form (ubiquinol), should therefore be substantially more effective than aqueous-phase singlet oxygen quenchers. This reframes the conventional antioxidant hierarchy: the target shifts from quenching the initial reactive species to interrupting the amplification cascade that converts a single photochemical event into widespread membrane damage. Formulations delivering lipophilic, membrane-partitioning antioxidants to the dermis may provide protection that is mechanistically orthogonal to, and synergistic with, UV filtration.

Strategy 3: support organelle-specific defense systems. The organelle-targeting evidence (Section 5) shows that mitochondrial and lysosomal integrity are the critical determinants of cell fate following photodamage. Photoprotection strategies could therefore aim to reinforce the specific defenses of these organelles. For mitochondria, this includes maintaining cardiolipin integrity through adequate supply of its biosynthetic precursors, supporting mitochondrial SOD2 activity, and preserving the mitochondrial membrane potential that drives cationic PS accumulation. For lysosomes, maintaining membrane integrity, preserving acidic conditions through V-ATPase support, and promoting efficient autophagic flux are essential for clearing the photodamaged material before it accumulates as lipofuscin. Agents that protect lysosomal membranes from photo-oxidative damage, such as carbohydrate-rich formulations, may help preserve lysosomal function under photostress.^155^ Additionally, pharmacological induction of autophagy through repurposed drugs such as metformin represents a complementary approach to enhance clearance of photodamaged organelles.^177^ Furthermore, melanophagy-oriented interventions may offer a complementary strategy to mitigate melanin-derived phototoxicity while preserving epidermal homeostasis.^169^ Pharmacological activators of Nrf2 (Section 6.2), including sulforaphane, dimethyl fumarate, and other electrophilic compounds, represent a particularly attractive strategy because they upregulate both mitochondrial and cytosolic antioxidant defenses simultaneously, enhancing the capacity of the cell to maintain eustress rather than tipping into distress.

Strategy 4: target the lipofuscin feed-forward cycle. The lipofuscin analysis (Section 7.1) identifies a self-amplifying damage loop that is arguably the single most important target for visible light photoprotection. Breaking this cycle requires intervention at one or more of its steps: reducing lipofuscin formation (by minimizing the oxidative damage and impaired autophagy that produce it), enhancing lipofuscin clearance (by promoting autophagy and lysosomal function), or reducing lipofuscin photoreactivity (by quenching the radicals it generates before they propagate). Autophagy-inducing compounds such as rapamycin analogues, trehalose, and spermidine have shown efficacy in reducing lipofuscin accumulation in cell culture models, though their dermatological application remains largely unexplored.^178^ Chelation of the labile iron within lipofuscin granules could reduce Fenton-mediated radical amplification. The combination of autophagy support with membrane-localized antioxidants addresses both the source and the consequence of the feed-forward cycle, offering a mechanistically rational approach to visible light photoprotection that has no precedent in current suncare formulations.

### 8.2 Visible light protection: addressing the spectral gap

Conventional sunscreen formulations are optimized for ultraviolet absorption and provide negligible protection above 400 nm and do not properly consider the different approaches to different skin types. Yet the endogenous photosensitizers described in Section 7, lipofuscin (broad absorption peaking at around 450 nm), melanin (absorption extending to 500 nm), riboflavin (absorption maximum at 450 nm), and protoporphyrin IX (Soret band at 405 nm, with additional Q-bands extending into the red), all absorb strongly in the visible range. This creates a spectral gap in current photoprotection: the wavelengths most effective at activating endogenous photosensitizers in dermal and epidermal cells are precisely those that sunscreens do not block.

The biological response to light is fundamentally determined by an individual's endogenous chromophore profile, which varies dramatically with skin type.^15^ For individuals with darker skin (Fitzpatrick IV-VI), the high concentration of melanin provides a substantial intrinsic barrier against UV-induced erythema and direct DNA damage, granting them an inherent SPF. However, this very melanin richness creates a distinct and paradoxical vulnerability: visible light becomes a dominant driver of cutaneous pathology. As extensively documented in the literature, pigmentation disorders such as post-inflammatory hyperpigmentation (PIH) and melasma are far more prevalent and severe in darker skin populations, largely because their melanocytes are more reactive to inflammatory stimuli and hormonal triggers, and are exquisitely sensitive to visible light.^179^ The mechanisms involve the activation of not only melanin precursors but also other endogenous photosensitizers like lipofuscin and flavins, which absorb strongly in the visible spectrum and can initiate a feed-forward cycle of pigmentation. Critically, conventional UV filters offer negligible protection against these wavelengths. Consequently, photoprotection for darker skin must be redesigned with visible light as the primary target. Furthermore, strategies must address the post-inflammatory component by incorporating membrane-localized antioxidants to quench the radical chain propagation in melanocytes and keratinocytes, thereby interrupting the cycle of hyperpigmentation triggered by visible light exposure. In lighter skin phototypes (Fitzpatrick I–II), where melanin concentrations are low, there is high photobiological risk of the development of acute erythema (sunburn) and direct DNA damage from UV-B, leading to photocarcinogenesis. For these individuals, visible light, particularly in the blue region, can initiate oxidative stress through melanin-mediated pathways, contributing to photoaging and potentially to the formation of “dark CPDs” *via* chemiexcitation.^166,180,181^ Therefore, for lighter skin, improved photoprotection must extend beyond high-SPF UV filters and should include antioxidants capable of intercepting the radical chain reactions.^182^

Iron oxide pigments represent the most established approach to visible light protection. Tinted sunscreen formulations containing iron oxides absorb across the visible spectrum and have demonstrated superior protection against visible light-induced hyperpigmentation compared to non-tinted formulations with equivalent SPF.^183^ However, not all iron oxide formulations provide equivalent protection, and the degree of protection depends on the specific iron oxide composition, particle size, and concentration.^184^ Critically, no standardized metric equivalent to SPF exists for visible light protection, creating a barrier to consumer understanding and product comparison.

Emerging approaches to visible light filtration include melanin-mimetic nanoparticles that replicate the broadband absorption and thermal dissipation properties of eumelanin (**Box 2**) in a synthetic, stable format; organic blue-light filters with absorption maxima tuned to the 400–500 nm range; and hybrid formulations combining UV filters with visible light absorbers and antioxidants. The development of biological blue-light filters faces a fundamental design challenge: the filter must absorb visible light efficiently (to prevent it from reaching endogenous chromophores) while dissipating the absorbed energy as heat rather than generating oxidants, essentially replicating the photophysics of eumelanin rather than pheomelanin. This is precisely the distinction between a physical quencher and a photosensitizer defined in Section 2: an effective visible light filter must absorb without sensitizing.

The lack of standardized testing methods for visible light protection represents a significant gap in regulatory science. A visible light protection factor (VLPF) analogous to SPF would require agreement on the action spectrum (weighting the contribution of different visible wavelengths to photodamage), the relevant biological endpoint (pigmentation, oxidant generation, or collagen degradation), and the measurement methodology (*in vivo*, *in vitro*, or spectrophotometric). The endogenous photosensitizer absorption spectra characterized in Section 7 provide a rational basis for defining the action spectrum: wavelengths weighted by the combined absorption of lipofuscin, PpIX, and flavins would capture the principal drivers of visible light photodamage in skin.

### 8.3 The photon paradox and personalized photoprotection

The convergence of PDT mechanistic insights with suncare science opens several research directions that could transform photoprotection from a purely photon-blocking strategy to a multi-targeted, biologically informed approach. The fundamental constraint, however, is that total photon exclusion is neither achievable nor desirable: skin biology depends on light.

#### 8.3.1 The photon paradox: why total filtration is biologically untenable

Conventional photoprotection treats all incident radiation as damaging, but skin cells actively use photons for essential signaling. UV-B drives cutaneous vitamin *D*_3_ synthesis from 7-dehydrocholesterol^185^ and triggers β-endorphin release through POMC expression.^186^ UV-A releases nitric oxide from cutaneous stores, lowering blood pressure independently of vitamin D or NOS activity.^187^ Perhaps most relevant to this review, visible light itself is a physiological signal in skin. Human epidermal keratinocytes and melanocytes express multiple opsins, with OPN3 (encephalopsin/panopsin) the most highly expressed in both cell types.^188^ OPN3 mediates blue-light-dependent melanogenesis in melanocytes through a calcium/CaMKII/CREB pathway that upregulates MITF and tyrosinase expression,^189^ and in keratinocytes it drives UV-A-induced supranuclear melanin cap formation, one of the skin's primary endogenous photoprotective responses.^190^ Blocking visible light entirely would suppress these opsin-mediated adaptive responses. This creates a paradox at the heart of photoprotection: the same wavelengths (particularly 400–500 nm) that are absorbed by endogenous photosensitizers to generate damaging reactive species are also required for opsin-mediated protective signaling. The problem cannot be solved by surface filtration alone.

#### 8.3.2 Endogenous photosensitizers compound the problem

Even if one were willing to sacrifice the beneficial photobiology, surface filtration still cannot neutralize photosensitizers already present within viable cells. Lipofuscin, the heterogeneous aggregate of oxidized lipids and proteins that accumulates in lysosomes during cellular aging, functions as a potent visible-light photosensitizer in human keratinocytes. UV-A exposure accelerates lipofuscin accumulation; the resulting lipofuscin-loaded cells generate singlet oxygen upon blue light excitation (peak ∼450 nm) and sustain oxidative DNA damage including Fpg-sensitive and Endo III-sensitive lesions.^17,18^ Melanin and its oxidation products similarly photosensitize visible-light-driven damage, and advanced glycation end products (AGEs) in dermal collagen represent a third class of endogenous photosensitizer whose accumulation increases with age, diabetes, and chronic UV exposure.^15^ Because these photosensitizers are intracellular, no surface-applied filter can prevent their excitation by the visible photons that must reach the skin for normal signaling. This is the mechanistic rationale for pursuing intracellular photoprotection strategies.

#### 8.3.3 Personalized risk stratification through autofluorescence

Non-invasive measurement of skin autofluorescence is already clinically available for AGEs: the AGE Reader excites at 370 nm and detects emission at 420–600 nm, with reference values established across age and disease states.^191^ Whether analogous non-invasive measurement of skin lipofuscin autofluorescence can be developed remains an open translational question; the spectroscopic characterization of keratinocyte lipofuscin (excitation maximum ∼450 nm, emission >515 nm, lifetime ∼1.7 ns) is well established *in vitro* but has not yet been demonstrated *in vivo* in human skin. If such a measurement became feasible, it could identify individuals with elevated endogenous photosensitizer burdens, typically older individuals, diabetics, or those with chronic sun exposure, and guide personalized recommendations for supplementary antioxidant and visible-light-filtering ingredients, shifting photoprotection from a one-size-fits-all model to a risk-stratified strategy.

#### 8.3.4 Combination strategies informed by mechanistic complementarity

For individuals carrying significant endogenous photosensitizer loads, the four strategies outlined in Section 8.1 address different steps in the photodamage cascade and are therefore mechanistically complementary rather than redundant. A comprehensive photoprotection formulation could combine (i) UV filters for UV-B and UV-A, (ii) visible-light absorbers such as iron oxides for the 400–500 nm range, which attenuate the most damaging wavelengths while still transmitting enough visible light for opsin signaling, and have demonstrated superior protection against visible-light-induced pigmentation compared with conventional mineral SPF 50+ sunscreens in randomized trials,^183,192^ (iii) membrane-localized chain-breaking antioxidants (α-tocopherol and ubiquinol) to interrupt lipid peroxidation propagation, and (iv) Nrf2-activating compounds to upregulate endogenous antioxidant defenses. Sulforaphane, a potent Nrf2 activator, has demonstrated photoprotective effects against UV-B-induced inflammation and sunburn in Nrf2-dependent fashion in murine skin, with protection mediated through induction of heme oxygenase-1 and phase II detoxification enzymes.^193,194^ Because each layer of protection addresses a distinct mechanistic target, their combination should in principle provide greater-than-additive benefit, although formal demonstration of synergy in a relevant photoprotection model is still lacking.

#### 8.3.5 Reverse PDT logic for protective formulation design

PDT research has developed sophisticated strategies for delivering photosensitizers to specific organelles using targeting moieties: triphenylphosphonium (TPP) cations for mitochondria, morpholine groups for lysosomes, and peptide sequences for various compartments. The same targeting chemistry could be applied in reverse, delivering antioxidants to the organelles most vulnerable to endogenous photosensitization. Mitochondria-targeted antioxidants such as MitoQ (ubiquinone conjugated to a TPP cation *via* a decyl chain) and SkQ1 (plastoquinone-TPP) accumulate in the mitochondrial inner membrane driven by the membrane potential, where they are reduced to their quinol forms by the electron transport chain and thereby scavenge oxidant species at the site of their generation.^195^ SkQ1 has demonstrated protective efficacy against UV-induced corneal oxidative damage in a rabbit model, reducing malondialdehyde levels and preserving antioxidant capacity.^196^ It is important to note that MitoQ and SkQ1 function primarily as recyclable oxidant scavengers that attenuate superoxide formation at Complex I and Complex III, rather than intercepting propagating peroxyl radicals in the manner of classical chain-breaking antioxidants like α-tocopherol. Their application to photoprotection, delivering recyclable antioxidant capacity to the organelle where cardiolipin and other mitochondrial lipids are most vulnerable to photosensitized oxidation, represents a direct translation of PDT targeting principles to suncare, although it remains at the preclinical stage.

### 8.4 Next-generation PDT agent design: from photosensitization to photoinitiation

For PDT itself, the insights of this review point toward a clear design strategy for next-generation therapeutic agents. Rather than maximizing singlet oxygen quantum yield in solution, the ideal agent should be optimized for membrane-contact photochemistry at the target organelle. Specifically, a therapeutic photosensitizer designed to function as a PI or PCI at the target membrane should possess sufficient amphiphilicity to partition deeply into organelle membranes, a reduction potential favorable for direct electron or hydrogen atom abstraction from unsaturated lipids, organelle-targeting moieties that direct accumulation to mitochondria, lysosomes, or both, and absorption in the therapeutic window compatible with tissue penetration. These criteria prioritize photoinitiation or photocatalytic initiation of lipid peroxidation chains at the target membrane over bulk singlet oxygen generation, reflecting the mechanistic shift from the traditional photochemical efficiency paradigm to the localization-and-contact paradigm developed throughout this review. This design philosophy does not diminish the clinical achievements of existing Type II-dominated agents such as porfimer sodium, temoporfin, and verteporfin, which have demonstrated therapeutic efficacy despite suboptimal membrane localization. Rather, it suggests that agents engineered for membrane-contact reactivity could achieve equivalent or superior tumor destruction at lower light doses and reduced off-target phototoxicity.

## 9 Future outlook

The mechanistic chain connecting photon absorption to cell fate can now be stated with considerable specificity: photosensitizer localization at organelle membranes enables contact-dependent Type I reactions that generate lipid radicals propagating self-sustaining peroxidation chains, producing truncated aldehydes that form transmembrane pores. The resulting organelle permeabilization activates regulated cell death through apoptosis, ferroptosis, pyroptosis, or autophagy-associated pathways, with dual-organelle targeting amplifying these effects synergistically. These same mechanisms operate during chronic sun exposure, where endogenous photosensitizers such as lipofuscin, protoporphyrin IX, pheomelanin, and flavins engage identical membrane permeabilization pathways under ambient visible light. The lipofuscin feed-forward cycle, in which photodamage impairs autophagy and impaired autophagy increases photosensitizer accumulation, provides a molecular mechanism for the progressive visible light vulnerability of aging skin that operates below the threshold of acute cell death but above the threshold of eustress.

The PS/PC/PI/PCI classification provides the mechanistic language needed to unify these phenomena. Recognizing that many biologically relevant photosensitizers function as photocatalytic initiators generating radical chains that amplify damage beyond the stoichiometric capacity of the absorber resolves longstanding puzzles in both fields: why singlet oxygen quantum yield poorly predicts PDT efficacy, why membrane-embedded photosensitizers with negligible singlet oxygen production outperform efficient generators in the aqueous phase, and why modest visible light doses produce disproportionate damage in lipofuscin-laden cells. This shared mechanistic vocabulary should accelerate cross-fertilization between PDT and photodermatology, two communities that have much to learn from each other.

The actionable outputs are symmetric. For PDT, design agents that function as photoinitiators or photocatalytic initiators at the target organelle membrane, maximizing membrane affinity and Type I reactivity rather than singlet oxygen yield, exploit dual-organelle targeting to activate synergistic death pathways and block autophagic rescue. For suncare:, move beyond photon blocking to a multi-layered strategy combining UV filtration, visible light absorption in the 400–500 nm gap, membrane-localized chain-breaking antioxidants, and organelle-support strategies that maintain autophagic flux and Nrf2-mediated defenses. The conceptual bridge between these fields has been missing. The tools to build on it already exist.

## Author contributions

Conceptualization: ELB and MSB; writing – original draft: ELB and MSB; writing – review & editing: ELB, WKM, RI, TTT, and MSB; visualization: ELB; project administration: MSB; supervision: MSB; funding acquisition: ELB and MSB.

## Conflicts of interest

There are no conflicts to declare.

## Data Availability

This review article does not contain original data. All information discussed is derived from previously published work cited in the references.
